# CIViCdb 2022: evolution of an open-access cancer variant interpretation knowledgebase

**DOI:** 10.1093/nar/gkac979

**Published:** 2022-11-14

**Authors:** Kilannin Krysiak, Arpad M Danos, Jason Saliba, Joshua F McMichael, Adam C Coffman, Susanna Kiwala, Erica K Barnell, Lana Sheta, Cameron J Grisdale, Lynzey Kujan, Shahil Pema, Jake Lever, Sarah Ridd, Nicholas C Spies, Veronica Andric, Andreea Chiorean, Damian T Rieke, Kaitlin A Clark, Caralyn Reisle, Ajay C Venigalla, Mark Evans, Payal Jani, Hideaki Takahashi, Avila Suda, Peter Horak, Deborah I Ritter, Xin Zhou, Benjamin J Ainscough, Sean Delong, Chimene Kesserwan, Mario Lamping, Haolin Shen, Alex R Marr, My H Hoang, Kartik Singhal, Mariam Khanfar, Brian V Li, Wan-Hsin Lin, Panieh Terraf, Laura B Corson, Yasser Salama, Katie M Campbell, Kirsten M Farncombe, Jianling Ji, Xiaonan Zhao, Xinjie Xu, Rashmi Kanagal-Shamanna, Ian King, Kelsy C Cotto, Zachary L Skidmore, Jason R Walker, Jinghui Zhang, Aleksandar Milosavljevic, Ronak Y Patel, Rachel H Giles, Raymond H Kim, Lynn M Schriml, Elaine R Mardis, Steven J M Jones, Gordana Raca, Shruti Rao, Subha Madhavan, Alex H Wagner, Malachi Griffith, Obi L Griffith

**Affiliations:** Department of Pathology and Immunology, Washington University in St Louis School of Medicine, St. Louis, MO, USA; McDonnell Genome Institute, Washington University in St Louis School of Medicine, St. Louis, MO, USA; Siteman Cancer Center, Washington University in St Louis School of Medicine, St. Louis, MO, USA; Department of Medicine, Washington University in St Louis School of Medicine, St. Louis, MO, USA; McDonnell Genome Institute, Washington University in St Louis School of Medicine, St. Louis, MO, USA; Department of Medicine, Washington University in St Louis School of Medicine, St. Louis, MO, USA; Department of Medicine, Washington University in St Louis School of Medicine, St. Louis, MO, USA; McDonnell Genome Institute, Washington University in St Louis School of Medicine, St. Louis, MO, USA; McDonnell Genome Institute, Washington University in St Louis School of Medicine, St. Louis, MO, USA; McDonnell Genome Institute, Washington University in St Louis School of Medicine, St. Louis, MO, USA; McDonnell Genome Institute, Washington University in St Louis School of Medicine, St. Louis, MO, USA; McDonnell Genome Institute, Washington University in St Louis School of Medicine, St. Louis, MO, USA; Canada's Michael Smith Genome Sciences Centre, Vancouver, BC, Canada; McDonnell Genome Institute, Washington University in St Louis School of Medicine, St. Louis, MO, USA; McDonnell Genome Institute, Washington University in St Louis School of Medicine, St. Louis, MO, USA; School of Computer Science, University of Glasgow, Glasgow, UK; Department of Medicine, Division of Medical Oncology, University Health Network, Toronto, Ontario, Canada; McDonnell Genome Institute, Washington University in St Louis School of Medicine, St. Louis, MO, USA; Department of Medicine, Division of Medical Oncology, University Health Network, Toronto, Ontario, Canada; Department of Medicine, Division of Medical Oncology, University Health Network, Toronto, Ontario, Canada; Charité – Universitätsmedizin Berlin, corporate member of Freie Universität Berlin and Humboldt-Universität zu Berlin, Berlin, Germany; McDonnell Genome Institute, Washington University in St Louis School of Medicine, St. Louis, MO, USA; Canada's Michael Smith Genome Sciences Centre, Vancouver, BC, Canada; Bioinformatics Graduate Program, Faculty of Science, University of British Columbia, Vancouver, BC, Canada; Department of Medicine, Washington University in St Louis School of Medicine, St. Louis, MO, USA; Caris Life Sciences, Phoenix, AZ, USA; Department of Medicine, Division of Medical Oncology, University Health Network, Toronto, Ontario, Canada; Department of Experimental Therapeutics/Department of Hepatobiliary and Pancreatic Oncology, National Cancer Center Hospital East, Kashiwa, Japan; Department of Medicine, Washington University in St Louis School of Medicine, St. Louis, MO, USA; Department of Translational Medical Oncology, National Center for Tumor Diseases (NCT) Heidelberg and German Cancer Research Center (DKFZ), Heidelberg, Germany; Department of Pediatrics, Baylor College of Medicine, Houston, TX, USA; Texas Children's Cancer Center, Texas Children's Hospital, Houston, TX, USA; Department of Computational Biology, St. Jude Children's Research Hospital, Memphis, TN, USA; McDonnell Genome Institute, Washington University in St Louis School of Medicine, St. Louis, MO, USA; Lassonde School of Engineering, York University, Toronto, Ontario, Canada; Department of Pathology, NYU Grossman School of Medicine, New York, NY, USA and Genetics Branch, National Cancer Institute, National Institute of Health, Bethesda, MD, USA; Charité – Universitätsmedizin Berlin, corporate member of Freie Universität Berlin and Humboldt-Universität zu Berlin, Berlin, Germany; Department of Medicine, Washington University in St Louis School of Medicine, St. Louis, MO, USA; Department of Pathology and Immunology, Washington University in St Louis School of Medicine, St. Louis, MO, USA; Department of Medicine, Washington University in St Louis School of Medicine, St. Louis, MO, USA; Department of Medicine, Washington University in St Louis School of Medicine, St. Louis, MO, USA; Department of Medicine, Washington University in St Louis School of Medicine, St. Louis, MO, USA; McDonnell Genome Institute, Washington University in St Louis School of Medicine, St. Louis, MO, USA; Mayo Clinic Florida, Jacksonville, FL, USA; Department of Pathology and Laboratory Medicine, Memorial Sloan Kettering Cancer Center, New York, NY, USA; Dana-Farber/Boston Children's Cancer and Blood Disorders Center, Boston, MA, USA; Department of Medicine, Division of Medical Oncology, University Health Network, Toronto, Ontario, Canada; McDonnell Genome Institute, Washington University in St Louis School of Medicine, St. Louis, MO, USA; Toronto General Hospital Research Institute, University Health Network, Toronto, Ontario, Canada; Children's Hospital Los Angeles, University of Southern California, Los Angeles, CA, USA; Department of Molecular and Human Genetics, Baylor College of Medicine, Houston, TX, USA; Division of Hematopathology, Department of Laboratory Medicine and Pathology, Mayo Clinic, Rochester, MN, USA; Department of Hematopathology and Molecular Diagnostics, The University of Texas MD Anderson Cancer Center, Houston, TX, USA; Division of Clinical Laboratory Genetics, Laboratory Medicine Program, University Health Network (UHN), Toronto, ON, Canada; Department of Medicine, Washington University in St Louis School of Medicine, St. Louis, MO, USA; McDonnell Genome Institute, Washington University in St Louis School of Medicine, St. Louis, MO, USA; McDonnell Genome Institute, Washington University in St Louis School of Medicine, St. Louis, MO, USA; Department of Computational Biology, St. Jude Children's Research Hospital, Memphis, TN, USA; Department of Molecular and Human Genetics, Baylor College of Medicine, Houston, TX, USA; Department of Molecular and Human Genetics, Baylor College of Medicine, Houston, TX, USA; International Kidney Cancer Coalition, Duivendrecht-Amsterdam, the Netherlands; Division of Medical Oncology and Hematology, Princess Margaret Cancer Centre, University Health Network, Sinai Health System, Division of Clinical and Metabolic Genetics, The Hospital for Sick Children, Ontario Institute for Cancer Research, Department of Medicine, University of Toronto, Toronto, Ontario, Canada; University of Maryland School of Medicine, Baltimore, MD, USA; The Steve and Cindy Rasmussen Institute for Genomic Medicine, Nationwide Children's Hospital, Columbus, OH, USA; Departments of Pediatrics and Neurosurgery, The Ohio State University College of Medicine, Columbus, OH, USA; Canada's Michael Smith Genome Sciences Centre, Vancouver, BC, Canada; Children's Hospital Los Angeles, University of Southern California, Los Angeles, CA, USA; Innovation Center for Biomedical Informatics, Georgetown University Medical Center, WA DC, USA; Innovation Center for Biomedical Informatics, Georgetown University Medical Center, WA DC, USA; The Steve and Cindy Rasmussen Institute for Genomic Medicine, Nationwide Children's Hospital, Columbus, OH, USA; Departments of Pediatrics and Biomedical Informatics, The Ohio State University College of Medicine, Columbus, OH, USA; McDonnell Genome Institute, Washington University in St Louis School of Medicine, St. Louis, MO, USA; Siteman Cancer Center, Washington University in St Louis School of Medicine, St. Louis, MO, USA; Department of Medicine, Washington University in St Louis School of Medicine, St. Louis, MO, USA; Department of Genetics, Washington University in St Louis School of Medicine, St. Louis, MO, USA; McDonnell Genome Institute, Washington University in St Louis School of Medicine, St. Louis, MO, USA; Siteman Cancer Center, Washington University in St Louis School of Medicine, St. Louis, MO, USA; Department of Medicine, Washington University in St Louis School of Medicine, St. Louis, MO, USA; Department of Genetics, Washington University in St Louis School of Medicine, St. Louis, MO, USA

## Abstract

CIViC (Clinical Interpretation of Variants in Cancer; civicdb.org) is a crowd-sourced, public domain knowledgebase composed of literature-derived evidence characterizing the clinical utility of cancer variants. As clinical sequencing becomes more prevalent in cancer management, the need for cancer variant interpretation has grown beyond the capability of any single institution. CIViC contains peer-reviewed, published literature curated and expertly-moderated into structured data units (Evidence Items) that can be accessed globally and in real time, reducing barriers to clinical variant knowledge sharing. We have extended CIViC’s functionality to support emergent variant interpretation guidelines, increase interoperability with other variant resources, and promote widespread dissemination of structured curated data. To support the full breadth of variant interpretation from basic to translational, including integration of somatic and germline variant knowledge and inference of drug response, we have enabled curation of three new Evidence Types (Predisposing, Oncogenic and Functional). The growing CIViC knowledgebase has over 300 contributors and distributes clinically-relevant cancer variant data currently representing >3200 variants in >470 genes from >3100 publications.

## INTRODUCTION

**Figure 1.**
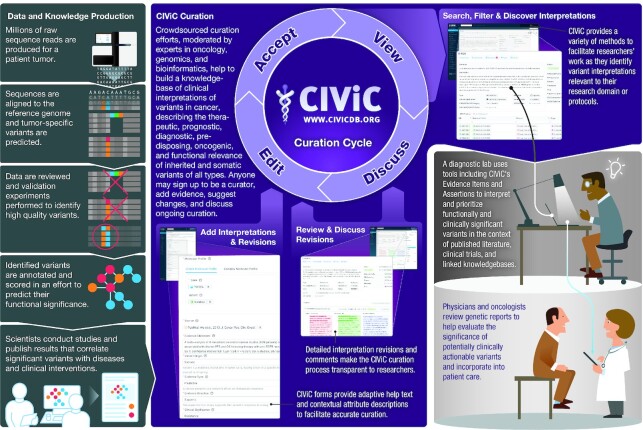
Overview of CIViC. Next generation sequencing of tumors has become integrated into clinical practice and the biomedical literature. CIViC curation of the biomedical literature involves cycles of curating, editing submitted content, moderation and eventually viewing of accepted knowledge by the public. The public in turn can give feedback through commenting and submitting revisions to curated content. The database of literature-curated evidence can help address the bottleneck problem which arises when large numbers of variants found in sequenced tumor tissue need to be characterized.

CIViC is an open-access, open-source, fully transparent knowledgebase for the expert crowdsourcing of Clinical Interpretation of Variants in Cancer (civicdb.org) first released in 2017 (1) (Figure 1). CIViC Curators extract detailed evidence of the clinical significance of variants in cancer from the peer-reviewed, published literature and selected conference abstracts to contribute to the resource. Crowdsourced contributions are moderated after submission by expert Editors who are familiar with CIViC standard operating procedures, have undergone training, and have field-relevant expertise (2).

CIViC was designed to encourage the development of community consensus by leveraging an interdisciplinary, international team of experts, collaborating remotely within a centralized curation interface. Curated variant interpretations are made available through a web interface (no login required) and a well-documented, modern application programming interface (API), under a public domain (CC0) dedication. All software is available on GitHub under an open source license (MIT).

The existing annotation bottleneck associated with variant interpretation is well described (3). The ever growing repertoire of variants associated with cancer has led to many falling outside of clinical guidelines, resulting in a need for resources to assist variant analysts, geneticists, and oncologists, among others. The expansion and improvement of platforms that can quickly and effectively incorporate genomics to guide the diagnosis, prognosis, and treatment of cancers is required to alleviate this bottleneck within precision oncology. Multiple efforts exist to address this need, but these efforts are siloed by limited accessibility and scope. The Variant Interpretation for Cancer Consortium (VICC) created a novel tool (4) to harmonize content from multiple disparate cancer variant evidence resources, including CIViC, OncoKB (5), JAX-CKB (6), the Precision Medicine Knowledgebase (7), and the CancerGenomeInterpreter Biomarkers Database (8), among others. The analysis of resources highlighted by that study identified major knowledge gaps, lack of overlap, and lack of standards for variant and disease naming. This illustrated the need for further evolution of cancer variant knowledgebases that support improved data standards, increased curation effort, and consensus guidelines for curation.

Since CIViC’s inception, the capabilities of the knowledgebase and interface have been expanded to meet the needs of the community through significant updates to the CIViC data model, curation practices, and knowledgebase content. In the process, the CIViC knowledgebase has taken on a unique role within the scientific community of variant interpretation, engaging with global stakeholders in academia, government, and industry.

Here, we describe the impact of the CIViC knowledgebase on the field through increased community activity, and updates to our data model and user interface, in response to changing curator needs and emerging guidelines for variant interpretation. Furthermore, we exemplify how these extensive improvements and increased international adoption of the CIViC knowledgebase has centralized curation and improved the distribution of curated cancer variant evidence.

### Scaling up curation through community engagement

**Figure 2.**
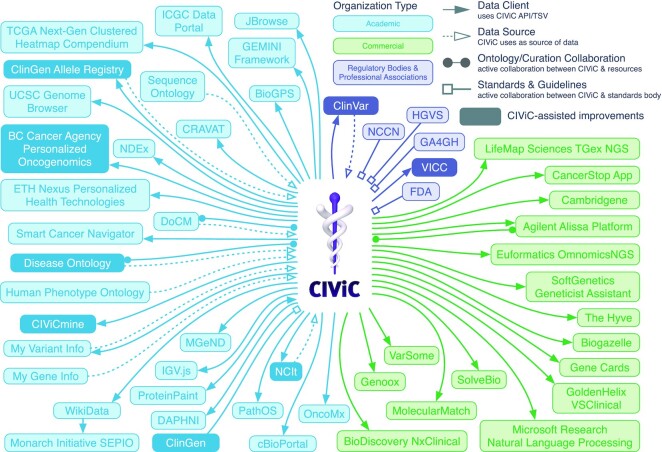
The CIViC Ecosystem. Community network showing engagement with the CIViC resource. Colors represent the Organization Type (Regulatory/Professional Associations, Academic, and Commercial) and connections indicate the type of interaction with the organization.

**Figure 3.**
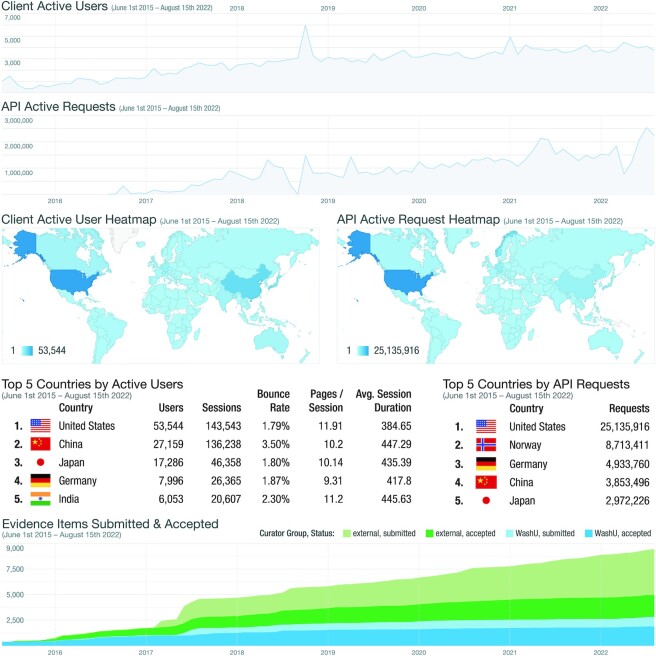
API and web usage statistics. Total engagements with the database are shown. Density plots (top) show client users accessing CIViC through the web interface (Client Active Users) and pull requests from the CIViC API since 2015. Heatmaps (middle) show the originating country (based on IP address) from users interacting with the web interface (left) and API (right). Activity from the top 5 countries is shown (middle), by active users and API requests. The single digit Bounce Rate indicates that over 97% of users engage with more than one page of CIViC content with users viewing approximately 11 pages per visit and spending on average 6.85 min in the web interface per visit. At the inception of the knowledgebase, initial contributions to the database (bottom) were performed by internal Curators (Washington University in St. Louis, WashU, dark and light blue). However, in 2017, external curation (Community, dark and light green) exceeded the internal contribution. To date, the gap between internal and external contribution continues to widen as new external users begin to adopt and contribute to the database. Curation activity has exceeded moderation activity of Editors, as represented by Accepted Evidence Items (dark green and blue areas) compared to Submitted Evidence Items (light green and blue areas).

Since its initial release, the CIViC knowledgebase has undergone rapid expansion and adoption supporting a broader ecosystem of cancer variant resources. Due to its unrestricted licensing and open API, CIViC data consumers are not required to register their use; therefore, the complete picture of CIViC’s impact cannot be determined. However, numerous established collaborations and self-identified data clients illustrate several types of integration and diverse stakeholder engagement (Figure 2) (4,9–11). Most groups and individuals that interact with CIViC consume the data without creating new content. Web users demonstrate international (more than 90,000 users from outside the United States) and active engagement with 8,706 sessions per month (Figures 2 and 3). Anyone can create a login and contribute, and those who comment, submit new content, or suggest revisions to existing content are referred to as CIViC Curators (*N* = 328 as of 15 August 2022, Supplemental Figure S1). The majority of these Curators are individuals from outside of the Washington University in Saint Louis (WashU) community (only 55 of 328 active Curators have a WashU affiliation, the site of CIViC’s initial development) (Supplemental Figure S2), and represent academic, governmental and commercial organizations (9,12). Curator contributions take many forms and require varying degrees of effort, which supports curation activities from individuals with a wide range of interests and expertise (Supplemental Figure S3). Among the more time-intensive activities is the curation and moderation of Evidence Items (EIDs). As the foundational unit of CIViC, EIDs associate a variant with a clinically-relevant interpretation derived from published biomedical literature. External contributions now greatly exceed internal contributions (Figure 3). Overall, CIViC EIDs have increased by 536% since initial publication (1), currently representing 3273 variants, 475 genes and 3192 sources for 340 different cancer types (Table 1, Supplemental Table S1). The most highly curated diseases according to accepted EIDs currently include: VHL, Lung Cancer, Colorectal Cancer, Leukemia (AML, CML, ALL), Breast Cancer, Melanoma, GIST, Ovarian Cancer, Head and Neck Cancer, and Brain Cancer (Glioblastoma).

**Table 1.** CIViC curation statistics from original publication to current

Category	1 December 2016	15 August 2022
Contributors	58	328
Total evidence items	1703	9127
Variants	731	3273
Sources	1076	3192
Total accepted evidence items	1678	4022
Drugs	291	488
Genes	283	475
Diseases	209	340

A subset of expert Curators (CIViC Editors; *N* = 32) are selected and trained to moderate submitted content and maintain the quality of the data in the resource, as previously described (1,2). Transparency of curation and moderation was improved through the addition of conflict of interest (COI) statements, which all Editors are required to complete annually. Editor functions are disabled for any Editor without an up-to-date COI. Editors are also barred from approving their own suggestions. This quality control policy requires all accepted content to have been reviewed by at least two Curators. Since the inception of CIViC, 3646 EIDs have been accepted by 15 WashU-affiliated Editors, and 405 by 17 community Editors (9.9%). A more recent snapshot of Editor activity (2022 year-to-date) shows 49.7% (80/161) of EIDs have been accepted by community Editors (Supplemental Figure S2b). These data illustrate the success of CIViC in engaging hundreds of outside users in the knowledge curation process.

### Guideline-driven evolution of CIViC variant tiering and classification

Several organizations have published guidelines for evaluating, interpreting, reporting, and cataloging evidence pertaining to cancer variants and their structured representation in databases (4,13–16). By supporting all variant types primarily associated with a single gene, including structural and copy number variants, CIViC is able to support the breadth of variants discussed in these guidelines (Supplemental Table S2). However, further support of the terminologies and criteria outlined in these recommendations required modifications to the CIViC data model. Some of these modifications have been recently described in detail elsewhere (2,17). In addition, the implementation of CIViC Assertions (Supplemental Figure S4) has permitted the integration of guidelines related to evidence aggregation and interpretation such as the classifications for somatic variants in cancer (AMP/ASCO/CAP) (13), Mendelian disorders (ACMG/AMP) (18) and oncogenicity (ClinGen/CGC/VICC) (16). CIViC Assertions aggregate EIDs for a given variant-disease or variant-disease-therapy combination to provide an overarching classification of clinical significance which reflects the state of the field, and are described in more detail in our curation standard operating procedure (SOP) (19). Assertions support different structured data fields than EIDs, including guideline-derived variant classification and evidence criteria, with specific fields and values dictated by the Assertion type. Additional Assertion-specific fields include National Comprehensive Cancer Network (NCCN) guidelines and Food and Drug Administration (FDA) approvals.

### CIViC provides the curation interface and serves as the database of record for the ClinGen Somatic Cancer CDWG

The Clinical Genome Resource (ClinGen) Somatic Cancer Clinical Domain Working Group (CDWG) facilitates the development of data curation guidelines and standards to determine the clinical significance of somatic alterations in cancer through structured collaboration (20). Over 200 multi-disciplinary experts in cancer biology, oncology, pathology, genetics, genomics and informatics have come together to create high-quality, clinically-significant somatic cancer Variant Assertions in the CIViC knowledgebase. Interfacing with the overall ClinGen consortium, the Somatic CDWG provides high level oversight and training, with 111 Curators and 11 Editors contributing to CIViC to date. Three Taskforces have been established—the Pediatric Cancer, Hematological Cancer and Solid Tumor Taskforces—whose efforts facilitate membership growth and targeted curation projects. Taskforces serve as incubators for the formation of Somatic Cancer Variant Curation Expert Panels (SC-VCEPs), the primary generators of the aforementioned high-quality clinical Assertions. SC-VCEPs undergo a four-step approval process adapted from ClinGen germline VCEP procedures (https://www.clinicalgenome.org/docs/clingen-variant-curation-expert-panel-vcep-protocol/) and develop granular interpretation specifications for their specific gene or disease focus. SC-VCEP variant classifications are publicly available as CIViC Assertions with supporting EIDs clearly displayed in the interface. ClinGen Somatic has directly curated 659 CIViC EIDs and 22 Assertions of clinical significance from 370 published papers into the CIViC knowledgebase. The Somatic Cancer CDWG utilizes CIViC’s Organization functionality (discussed in more detail below) to collate and track efforts within the CIViC knowledgebase. The goals of the ClinGen Somatic CDWG and CIViC strongly align, where each group plays complementary roles. By supporting the efforts of ClinGen Somatic, CIViC gains high-quality content and directly participates in ongoing guideline development while providing widespread dissemination of ClinGen curated content. This partnership is a critical collaboration for CIViC to diversify the expertise of its Curators, Editors, and the focus of the knowledgebase.

### Promoting curation transparency in CIViC

In 2018, the FDA announced a mechanism for recognition of public human genetic variant databases (21). A key criterion in the FDA recognition of genetic databases is transparency and public accountability, including description of expert panels and their members (21). To align with these guidelines, we have implemented Editor COI statements, a formal SOP (2), and the Organizations feature in CIViC. Organizations have individual pages in CIViC that display membership, summary statistics, and an activity feed which transparently displays member contributions. Upon request, the CIViC team assigns Curators to their specific Organizations and Sub-Organizations (Supplemental Figure S5). Organization-specific activity is tracked as each action performed by a Curator is tagged with their assigned Organization; or, if the Curator is associated with multiple Organizations (or Sub-Organizations), they can select the Organization best associated with an individual action from a drop down list. Currently, CIViC features eleven parent Organizations, the largest of which is ClinGen with 113 members and 7 Sub-Organizations (https://civicdb.org/organizations/2/members).

### Collaboration-driven evolution of the CIViC data schema

The evolution of the CIViC data schema has been as community-driven as the curation itself. Developments have ranged from major overhauls to support emergent guidelines, to adding small use-case-specific features to support external collaborations (Supplemental Figure S6). To obtain feedback and implement changes, curators and developers are routinely engaged through biennial, in-person Hackathon and Curation Jamborees, briefly outlined in the Supplementary Information. Specific examples of community-driven features are described below. Results of our first Hackathon and Curation Jamboree led to the development of a new Evidence Type (Predisposing Evidence) described elsewhere (17,19) and a fruitful collaboration with *VHL* experts for descriptive integration of variant and case-level data related to cancer predisposition syndromes (Supplemental Figure S7) (22). To achieve this, Human Phenotype Ontology (HPO) (23) terms were added as a new field to EIDs. HPO terms permit the tagging and later searching of the underlying phenotypes associated with variants described in the literature. In alignment with ClinGen and germline-focused VCEPs, curators of germline cancer predisposition variants are encouraged to make use of the ClinGen Variant Curation Interface (VCI) (24). However, support for germline curation in CIViC facilitates integrative interpretation of germline and somatic variants, and CIViC can also serve as a pre-VCI curation platform for germline VCEPs. CIViC Variant Evidence can be imported into the VCI through the LinkedDataHub (https://ldh.clinicalgenome.org/). At another Hackathon, we also worked on identifying an appropriate ontology to support Drug annotations for Predictive EIDs. At the inception of CIViC, no single ontology encompassed the breadth of drugs and treatments entered into CIViC (from preclinical investigational compounds to FDA-approved therapies), while reducing redundancies by supporting sufficiently curated names and aliases. The Hackathon working group proposed and began implementation of a tiered approach using the NCI Thesaurus (NCIt) (25) as the main source for drug concepts. We normalized 79% of existing Drugs in CIViC in our initial attempt and now use this ontology to automatically search for and normalize new content. To address terms not currently represented in NCIt, we allow non-NCIt Drugs to be entered, and through a more direct collaboration with NCIt, we curate and submit these terms to NCIt for integration on an ongoing basis, enriching both resources (Supplemental Figure S8). Hackathon events have also led to custom data formats and collaborative development to incorporate CIViC data into external resources such as NDEx (26), myvariant.info (27), WikiData (28), and openCRAVAT (29).

CIViC collaborated with the ClinGen Somatic Cancer CDWG and Human Disease Ontology to address the underrepresentation of pediatric cancer variants in interpretation resources. A tagging system for EIDs with pediatric data was developed utilizing HPO age of onset terms in the Associated Phenotypes field to enhance these pediatric cancer curations and their public dissemination. Pediatric cancer EIDs are tagged with onset terms that fall under the pediatric or young adult onset designations. To provide more granular curation of variants in the young adult range, we suggested new ontology terms to further segregate this onset range. These terms have been added by the HPO. Consistent feedback and submissions to resources we use, such as the NCIt (25), Human Disease Ontology (30) and ClinGen Allele Registry (31), promotes a collaborative ecosystem and provides a direct conduit for expert feedback from the needs of the CIViC community to these resources.

Through collaborations with the Variant Interpretation for Cancer Consortium (VICC; cancervariants.org) and ClinGen Somatic Cancer CDWG, we identified the need to curate evidence pertaining to a variant's impact on protein function or cellular properties. Large-scale genomic assays designed to describe the function of numerous variants allow for the evaluation of rare variants through comparison to established hotspot or targetable counterparts in the same gene (32). CIViC has set out to more clearly categorize variants based on their ability to induce measurable protein and cellular changes, by modifying the recently described Functional Evidence Type (2) to accommodate the creation of the Oncogenic Evidence Type. Both are described in more detail in the following text with additional examples available in the Supplementary Information.

### Expansion of the CIViC data model to include Functional and Oncogenic Evidence

The Functional Evidence Type strictly represents the variant's impact on protein function independent of disease context. EIDs of the Functional Evidence Type were designed to support fundamental genetic concepts introduced by Müller's Morphs (33), which include gain of function (hypermorphic), loss of function (amorphic), unaltered function (isomorphic), dominant negative (antimorphic), neomorphic, and unknown function (Supplemental Figures S9 and S10). Full inclusion of Müller's Morphs allows for more granular representations of protein level effects than those offered by most other resources, which are often limited to gain and loss of function. Functional genomics studies have also been specifically designed to query variants for neomorphic and dominant negative properties, which can drive different phenotypic effects and alter recommended treatment courses (34–36). Our new and expanded structure of the Functional Evidence Type thus provides the capacity for a thorough categorization of functional genomic results.

The new Oncogenic Evidence Type enables curation of variant interpretations related to the development and progression of cancer, as defined in the Hallmarks of Cancer (37). More specifically, Oncogenic Evidence describes a variant's role in influencing cancer development through sustaining proliferative signaling, resisting cell death, enabling replicative immortality, etc, rather than the variant's impact on protein function captured in Functional EIDs. Oncogenic EIDs may also be used to demonstrate that a variant has properties similar to another variant in the same gene which is approved for targeted therapy (38). Oncogenic properties are often cell-type dependent, so we require this Evidence Type to be associated with a Disease (39). CIViC Oncogenic EIDs may be used in the assessment of variants under professional society guidelines, including as supporting evidence for somatic clinical significance (13) and the recently published guidelines for oncogenic classification (Supplemental Figure S11) (16). Many in the CIViC community contributed to these recommendations for somatic variant oncogenicity, and we developed CIViC Oncogenic Assertions to incorporate those guidelines and further support the curation activities of ClinGen Somatic Variant Curation Expert Panels (SC-VCEPs).

### Introduction of new evidence sources to CIViC

Community engagement and feedback has emphasized the need for supporting curation of abstracts from national meetings where clinical trial results are presented. These often represent the most current results available and may include interim or final clinical trial results that will go unpublished. An evaluation of clinical trial results for breast, lung, colorectal, ovarian, and prostate cancers reported in abstracts from annual ASCO meetings (years 2009–2011) showed that 39% of findings remain unpublished 4–6 years later (40). Failed clinical trials often provide pertinent information for variant interpretation, but are less likely to be ultimately published. In other cases, regulatory approvals may be based, in part, on data only available in conference proceedings. To address the need for the curation of information derived from ASCO meetings, CIViC has augmented the accepted Source Types to support ASCO abstracts (Supplemental Figure S12). Curation procedures recognize this information should be used with caution given the limited access to detailed methodology, and that curation should only reflect the available data. Unfortunately, licensing restrictions and limited computational accessibility of content from additional peer-reviewed national meeting abstracts remains challenging for broader implementation, though we continue to pursue integration of other knowledge Source Types.

### Extension of CIViC software to highlight and integrate other variant resources

In addition to curation-driven collaborations, we continue to expand our software development collaborations. Manually providing depth and breadth of coverage of the ever-expanding biomedical literature is challenging for highly specialized curation tasks, such as identifying relevant cancer variants. Comparisons of cancer variant knowledgebases, including CIViC, have demonstrated a surprising dearth in publication overlap between key knowledgebases (4,41). To address this gap, colleagues at Canada's Michael Smith Genome Sciences Centre at BC Cancer leveraged experienced CIViC Editors to train a natural language processing model called CIViCmine to identify high-priority publications for CIViC and other cancer variant knowledgebases (http://bionlp.bcgsc.ca/civicmine/) (10). Ongoing efforts continue to expand the functionality and improve the integration of CIViCmine with CIViC. For instance, to aid the efforts of the ClinGen Somatic Pediatric Taskforce the CIViCmine resource is improving its coverage of underrepresented pediatric-associated clinical interpretations to enable discovery and extraction of relevant pediatric information from the literature.

Other critical collaborative projects have been expanded by outreach to other resources. The incorporation of the ClinGen Allele Registry (31) automatically connects manually curated (genomic) CIViC Variant Coordinates to their preferred genome build or transcript reference by using the Allele Registry link on CIViC Variant pages or by the Canonical Allele ID available through the CIViC API. Users evaluating variants via the ClinGen Allele Registry are similarly offered links back to CIViC. Analogous collaborations with the developers of St. Jude's ProteinPaint tool (42) have led to bidirectional links from CIViC to ProteinPaint, providing a visual representation of Variants in CIViC with curated coordinates alongside other key variant datasets such as COSMIC (43) and ClinVar (44). ProteinPaint users are directed in the interface to curated CIViC EIDs for their variants of interest. Additional collaborative products that have come from the CIViC knowledgebase can be found in Supplemental Table S3.

### CIViC as an educational and training resource

An increasing demand on the scientific community is the education and training of the next generation of biocurators, variant analysts, and geneticists (45–47). By making CIViC available without any installation requirements beyond a web browser and permitting any registered user to be a CIViC Curator, CIViC’s curation and variant interpretation interface has a low barrier for access, which proves useful in educational settings and workshops. Features such as the Source Suggestions queue (https://civicdb.org/curation/queues/pending-sources) (Supplemental Figure S12) provide a pre-selected list of potential PMIDs for curation that can be searched by Gene, Variant, Disease or publication year. To incentivize trainee activity, badges are awarded for various CIViC activity milestones (Supplemental Figure S5c). Training in CIViC curation promotes direct engagement with the clinical literature, develops skills in extracting evidence from the published literature, and provides interaction with clinical experts through the interface independent of time zone or physical location. Through summer internship programs, research collaborations, and formal courses, CIViC has been used as part of the training for undergraduate and graduate students with an interest in oncology or research in genetics. An open but rigorous variant interpretation resource such as CIViC can facilitate community engagement and provide mechanisms for the education of contributors, which ultimately produces higher quality contributions while supporting the advancement of the community at large.

### Improvements to the CIViC user experience

As the CIViC data schema and connections have expanded, additional documentation for Curators and software developers was necessary. Migration of our help documentation to a dedicated interface (hosted by readthedocs) lowers the maintenance cost for this documentation, a much-needed improvement that coincided with the development of a formalized curation SOP (2). Additional warnings and default page states have been introduced, which have become mainstays of the CIViC user experience, to emphasize higher-quality content and notify users of pending changes or unmoderated content. We have also provided faster, lower burden curation tasks, such as Flags to quickly draw Editor and community attention to potential inaccuracies (Supplemental Figure S3), Source Suggestions to recommend content for curation (Supplemental Figure S12), improved search functions (Supplemental Figure S13), and a redesign of the Variant interface (Supplemental Figure S14). Bioinformaticians and developers have taken advantage of the ease of CIViC’s API for integrations, with multiple groups having integrated CIViC without any direct interaction with our team (Figure 2). In addition to the API, data releases are now made available through monthly and nightly TSV and VCF files (https://civicdb.org/releases). TSV files are available for each of the major CIViC entities (EIDs, Assertions, Variants, and Genes). VCF files summarize CIViC Variants with curated Representative Coordinates and include annotations with data of submitted and accepted EIDs and Assertions linked to each Variant. These VCF files can be used to annotate patient variant calls with data available from CIViC for rapid clinical variant interpretation. For more programmatic approaches, CIViCpy (11), a software development kit enabling advanced CIViC queries, was developed for users familiar with Python.

### Improvements to CIViC user interface and API

In order to better support the continued evolution of the resource, significant improvements to the entire CIViC technical stack were performed. The frontend has been entirely redesigned to increase information density and discoverability (Supplemental Figures S13 and S14). Among the highlights of the new interface are dedicated pages for concepts linked to EIDs (such as NCIt Drug and Sequence Ontology Variant Type terms) and at-a-glance popover summaries of all CIViC entities. These changes provide users with more ways to browse and contextualize the knowledgebase. A new icon system was developed for all CIViC entities to allow users to more easily and quickly process the information presented (Supplemental Figure S6). On a technical level, the redesigned frontend has been written in Typescript and the new API follows the GraphQL specification. This combination allows the knowledge model to be more effectively expressed and validated in the type system of the application itself which eliminates certain types of errors, allows for strict validation of the data entered into CIViC, and brings the actual implementation in closer alignment with the knowledge model. The new API also allows for more efficient, performant queries for our data clients and users and expands the types of integrations that are possible to build with CIViC.

## SUMMARY AND FUTURE DIRECTIONS

As sequencing of cancers is increasingly used in routine patient care, more variants are discovered and the need for curated information to guide clinical variant interpretation continues to grow. Challenges to variant interpretation are further compounded by the exponential growth in medical literature, which greatly surpasses the ability of any one institution or group to assimilate. This bottleneck emphasizes the need for both open-access resources and community-generated contributions (17). By creating a resource with a low barrier to entry paired with expert moderation, CIViC promotes community engagement with the potential to scale with the medical literature. CIViC has made a significant contribution to this effort, inspired by widely-adopted bioinformatic tools used in sequence analysis, by developing an open-access and programmatically-accessible resource.

Since its inception, the CIViC project has seen considerable adoption from a growing community and substantial development of the knowledge model and user interface (Supplemental Table S4). As public contributions to CIViC have increased in number to outpace internal contributions from the CIViC team, the need for increased Editor capacity has become clear. Subsequently, Editor training and materials have been expanded. Assertions were developed in CIViC as a new curated data entity, which summarize evidence for a variant, apply a state-of-the-field clinical significance evaluation, and incorporate published guidelines for variant tiering (13,18). CIViC entered into collaborations with outside groups, notably the ClinGen Somatic Cancer CDWG, and in response developed Organizations to enable group tracking of curation conducted within a formal expert panel setting. Evidence Types for functional, oncogenic, and predisposing data were added, extending the data model to cover published guidelines (16,18) and supporting the collaborator-led incorporation of a large *VHL* variant dataset into CIViC (17,22). Further collaborative work resulted in the integration of CIViC into the broader community of variant interpretation resources and the development of the curation enhancement tool, CIViCmine. Many of these changes were reflected in updates to the user interface, culminating in the release of an updated version. Together, these improvements have broadened the scope, reach, and impact of the CIViC project.

The CIViC Ecosystem relies on the considerable volunteer efforts of Curators and Editors, user feedback, and integration of complementary resources. In turn, CIViC contributes to the ongoing development of this community through improved open-access data distribution, creation of educational materials, and providing feedback and curation to external resources. The acknowledgement and support of this community is critical to CIViC’s success. This work remains unfinished and there is a critical need for more expert Editors to moderate the contributions of Curators and keep pace with the ever expanding medical literature. The use of the CIViC platform for curation enables expert working groups from widely disparate time zones to collaborate asynchronously. However, despite engaging a diverse team of Curators, ongoing efforts are needed to engage not only more community Editors, but greater Editor representation outside of North America and Europe. Interested individuals can find more information on joining the CIViC community online at civicdb.org.

The CIViC platform provides a mechanism for variant evidence curation that supports computability and the FAIR principles (48), but also provides the flexibility for curators to use descriptive free-text variant names and aliases as needed where existing variant representation and nomenclatures fall short. The CIViC documentation covers conventions for variant naming to promote consistency among common variant types, including compound variants, codon- and exon-level variants, fusions, and more (https://civic.readthedocs.io/en/latest/model/variants/name.html). However this flexibility can hinder interoperability and automated concept matching. Furthermore, while CIViC EIDs try to capture the essential claims and intent of study authors, they do not provide primary analysis or reinterpretation of underlying data. Such claims may be highly context-specific, with varying degrees of subjectiveness and robustness. Consequently, the content of CIViC should always be interpreted carefully, in the context of other information in the field. For example, the p16 protein encoded by the *CDKN2A* gene has been used as a surrogate of HPV infection (49), and ‘p16 expression’ is a supported variant under *CDKN2A*, but expression level is subject to the variability in cutoffs and attention to detail of the article from which the data is derived.

As an open resource, CIViC does not direct what a Curator or Editor works on, as this decision is solely the choice of the individual. This has led to variable levels of completeness of curation when comparing genes and diseases. However, the collaboration with ClinGen Somatic has broadened the expertise of our Curators and diversified the scope of information entered and reviewed in the knowledgebase. Data provenance of all entries and actions in CIViC along with the exclusive Editor selection process and intense Editor training promote the integrity of our knowledgebase. The openness and flexibility of the CIViC data and curation model is not without pitfalls and caveats; however, the broad impact of this resource is demonstrated by the community's adoption and contributions.

Development work continues to address more complex issues, such as: variant relationships and combinations with clinical significance, integration of NLP-based resources that support discovery and curation, and support for large-scale variants that impact more than one gene. Such changes require substantial development efforts to adapt the database schema and the user interface to provide visual representations of this complex data and support more complex search capabilities. As this resource expands, we are committed to ensuring this work remains free and open to all, without fees or restrictions.

## DATA AVAILABILITY

CIViC data is available under a creative commons CC0 (public domain dedication) and can be viewed on https://civicdb.org. All underlying code is available on GitHub under an MIT license (https://github.com/griffithlab/civic-v2). Data is available via API (https://griffithlab.github.io/civic-v2/) and regular releases in TSV and VCF format (https://civicdb.org/releases/main). Additional documentation and help can be found in our help documentation: https://civicdb.org/pages/help.

## Supplementary Material

gkac979_Supplemental_File

## Notes

**Disclaimer:** The opinions expressed in this article are the author's own and do not reflect the view of the National Institutes of Health, the Department of Health and Human Services, or the United States government.
