# Supplementary material for: CIViCdb 2022: evolution of an open-access cancer variant interpretation knowledgebase
Source: Nucleic Acids Res. 2022 Nov 14;51(D1):D1230–41. doi: 10.1093/nar/gkac979 (PMC9825608; doi:10.1093/nar/gkac979)
Supplement: gkac979_Supplemental_File [file gkac979_supplemental_file.pdf]

Supplementary Information 1
CIViC Hackathon and Curation Jamborees 2
Functional and Oncogenic Evidence Examples 2
Relevant links to resources mentioned in the main and supplemental text 3

Supplementary Figures 4
Supplemental Figure 1. Growth of Curator base and acquisition of new curators 5
Supplemental Figure 2. Moderation activity by contribution source 5
Supplemental Figure 3. Range of curation activities within CIViC 6
Supplemental Figure 4. Example of a CIViC Assertion 7
Supplemental Figure 5. Curator incentivization through Organizations and Badges 8
Supplemental Figure 6. CIViC Data Model 9
Supplemental Figure 7. Germline and somatic VHL variants with Evidence Items and associated Human Phenotype Ontology terms 10
Supplemental Figure 8. New ‘Add Drug’ Interface 11
Supplemental Figure 9. Mapping of CIViC Functional Evidence to the classical genetic concepts of Müller’s Morphs 12
Supplemental Figure 10. Functional Evidence Item examples 13
Supplemental Figure 11. Oncogenic Evidence Item examples 14
Supplemental Figure 12. Integration of Sources to CIViC over existence of the knowledgebase 15
Supplemental Figure 13. Improved search capabilities 16
Supplemental Figure 14. Variant interface, then and now 17

Supplementary Tables 18
Supplemental Table 1. Diseases associated with 10 or more accepted Evidence Items 19
Supplemental Table 2. Types of Variants contained in CIViC as represented by assigned Sequence Ontology terms 20
Supplemental Table 3. Peer-reviewed publications associated with CIViC collaborations 21
Supplemental Table 4. Highlights of CIViC project development and community engagement since initial publication 22

Supplementary Information

CIViC Hackathon and Curation Jamborees

These CIViC-organized events are open to the community and occur in the style of an "unconference" where topics are driven by attendees(1). Attendees freely join breakout sessions and pursue topics of their choice while bringing back results or conclusions to the group as a whole at the end of the day or as part of the final session. To maximize the involvement in groups and presentations as well as interactions amongst participants, the attendee list is kept relatively small (registration capped at <50) and involves a few scheduled social events and meals over the 2-3 day period. The event planned for 2021 was canceled due to considerations associated with the spread of SARS-CoV-2.

The first event was held at the Netherlands Cancer Institute (NKI) Nov 30 - Dec 2, 2016 as part of the "NGS in Molecular Pathology Symposium". The second was held at Scripps Research Institute, La Jolla, CA Oct 15 - Oct 16 2018, as an adjunct meeting ahead of the American Society of Human Genetics (ASHG) annual meeting. These meetings focused on topics in the CIViC open-source code base, curation in CIViC, and issues of curation for the cancer variant community in general. Specific topics and breakout sessions from these meetings can be viewed on GitHub (github.com/griffithlab/civic-meeting). For example, topics in 2016 included methods of incentivizing curators, incorporating pharmacogenomic and predisposing biomarkers in CIViC, and capturing the complexities of therapeutic responses in preclinical models in a variant knowledgebase. Topics in 2018 included exporting CIViC data to variant call format (VCF) files, making CIViC networks available in NDEx, systematic evaluation of somatic variant oncogenicity, and best practices in annotating copy number variants (CNVs) and fusions in knowledgebases. These sessions have brought together individuals with a wide range of experience levels to produce collaborative efforts such as resource integrations (e.g., NDEx, CRAVAT, NCI thesaurus), data modeling and exporting, collaborative curation efforts (e.g., VHL as described in the main text and building on collaborator's efforts(2)) and involvement in external organizations (e.g., Somatic Cancer Clinical Domain Working Group [https://clinicalgenome.org/curation-activities/somatic/](3) and the Variant Interpretation for Cancer Consortium's Knowledge Curation and Interpretations Standards working group [cancervariants.org/wg/kcis/]).

Functional and Oncogenic Evidence Examples

Functional Evidence Items (EIDs) in CIViC are designed to capture evidence describing altered protein function due to the presence of a variant. This Evidence Type does not capture variant effects on cellular behavior, or assess the status of the variant as an oncogenic driver, and does not take disease into account. Functional EIDs will generally describe the results of *in vitro* studies (**Supplemental Figure 8**).

Oncogenic EIDs also primarily capture *in vitro* evidence, but this Evidence Type focuses on the downstream oncogenic effects of the alteration, regardless of the type of underlying functional change (e.g., loss of function, dominant negative) that led to those oncogenic effects (**Supplemental Figure 9**). This Evidence Type was designed to improve CIViC support of recently published oncogenicity guidelines(4). Structured fields in the Oncogenic EID allow for an annotation of support, or lack of support, for an oncogenic effect of that variant. Oncogenic EID curation is derived from the associated publication or meeting abstract's accounts describing evidence for the variant's potential in driving cancer. The notion of cancer driving potential which is used in Oncogenic EID curation is related to the Hallmarks of Cancer(5) (e.g. Resisting cell death, Evading growth suppressors, Deregulating cellular energetics, etc).

CIViC has a mechanism to capture the observation of a variant in a somatic tumor for assessment as a potential driver. Somatic tumors often harbor many variants — ranging from oncogenic to passenger variants,

with many variants being rare or having uncertain significance. Collecting information describing the potential oncogenicity of rare variants in genes with well-established oncogenic roles can be particularly important in some cases (**Supplemental Figure 11**). For example, common oncogenic KRAS variants are found at codons 12, 13, 61, and 146, but rare variants in other codons also occur. In these cases, information regarding oncogenicity of these rare variants is important because in colorectal cancer activating KRAS variants are associated with resistance to EGFR inhibitors. CIViC contains Oncogenic EIDs (**Supplemental Figure 11b**), which describe rare activating in-frame insertions found in colorectal cancer patients, near the common G12 driver variant site(6). *In vitro* and *in vivo* studies described in these EIDs establish the oncogenic role of these rare variants and could be used to influence clinical decision making. A formal discussion of inferences made for drug treatment based on similar oncogenic properties of different variants is outlined in the ESMO Scale for Clinical Actionability of molecular Targets (ESCAT)(7).

However, not all variants observed in somatic tumors meet the clinical threshold for inclusion in CIViC. To avoid the accumulation of variants with no established role in cancer, simple observations of a variant in a cancer are not entered into CIViC without substantial additional evidence or support. Therefore, Oncogenic EIDs documented in CIViC should meet stricter criteria for clinical importance, which are outlined in our help documentation (docs.civicdb.org).

Relevant links to resources mentioned in the main and supplemental text

CIViC interface

- civicdb.org

CIViC help documentation

- Docs.civicdb.org

Variant Interpretation for Cancer Consortium

- https://cancervariants.org/

ClinGen Somatic Cancer Clinical Domain Working Group (SC-CDWG)

- https://clinicalgenome.org/curation-activities/somatic/

ClinGen SC-VCEPs four-step approval process

- https://www.clinicalgenome.org/docs/clingen-variant-curation-expert-panel-vcep-protocol/

ClinGen Linked Data Hub

- https://ldh.clinicalgenome.org/

ClinGen Variant Curation Interface

- https://curation.clinicalgenome.org

CIViCmine

- http://bionlp.bcgsc.ca/civicmine/

Example CIViC Organization with Sub-Organizations (ClinGen)

- https://civicdb.org/organizations/2/members

CIViC API documentation

- https://griffithlab.github.io/civic-v2/

CIViC data releases (VCF, TSV)

- https://civicdb.org/releases

CIViC Source Suggestions queue

- https://civicdb.org/curation/queues/pending-sources

CIViC YouTube Channel (CIViCdb)

- https://www.youtube.com/channel/UCtZ14M7ZNmMDivSJzaHO8BQ

CIViC Twitter Account

- https://twitter.com/CIViCdb

Supplementary Figures

Supplemental Figure 1. Growth of Curator base and acquisition of new curators

The growth of CIViC's Curator base since its initial launch in 2015. Curators are defined as users who created an account and performed Curator actions in the interface such as adding Evidence Items, suggesting changes, commenting, or adding Source Suggestions. Total contributing Curators are shown in purple. White dots represent the number of new Curators added in each calendar month.

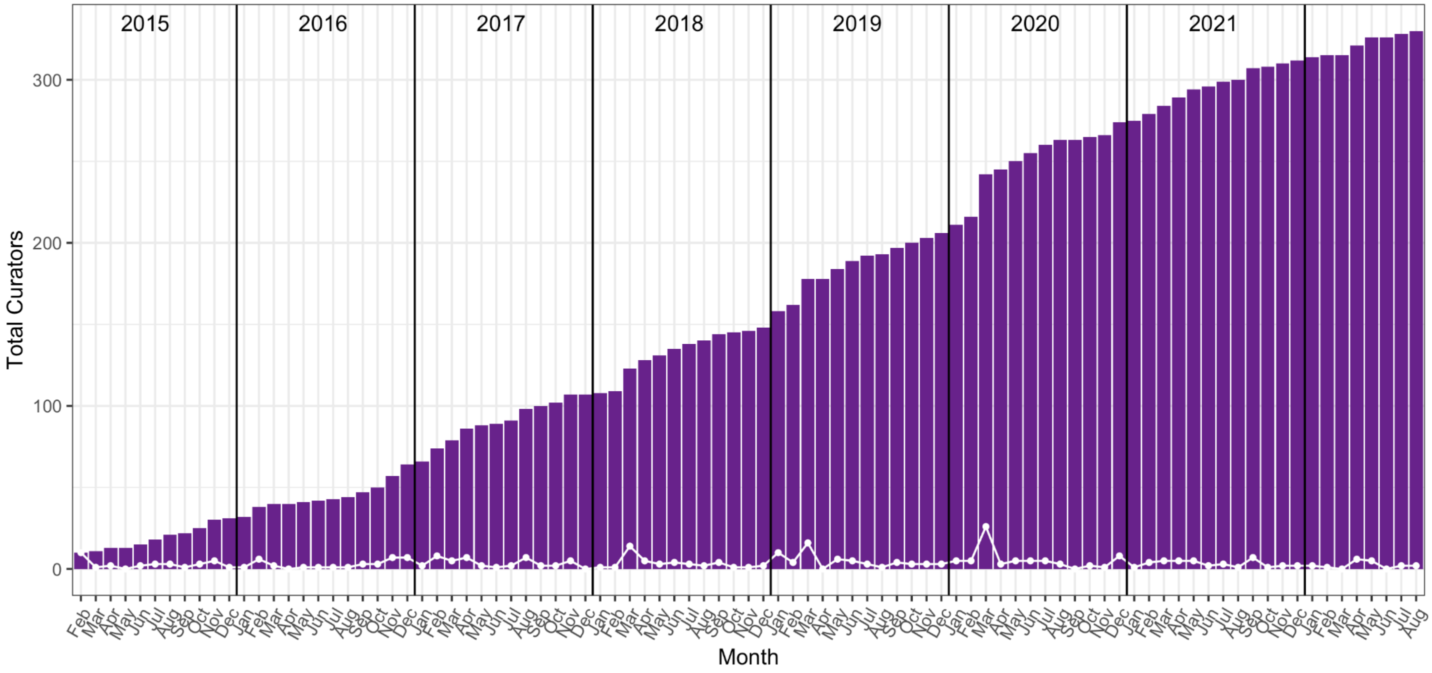

Supplemental Figure 2. Moderation activity by contribution source

a) The current affiliations of Curators (only those who have performed curation activities, excluding Editors, N=296) and Editors (N=32) working in the CIViC interface. **b)** After the initial launch, community Curators (external to Washington University in St Louis, WashU) were identified, trained and promoted to participate in moderation activities including accepting Evidence Items. As one measure of Editor activity, Evidence Item moderation in the form of acceptance events by internal Editors (WashU) vs external Editors (Community) are shown.

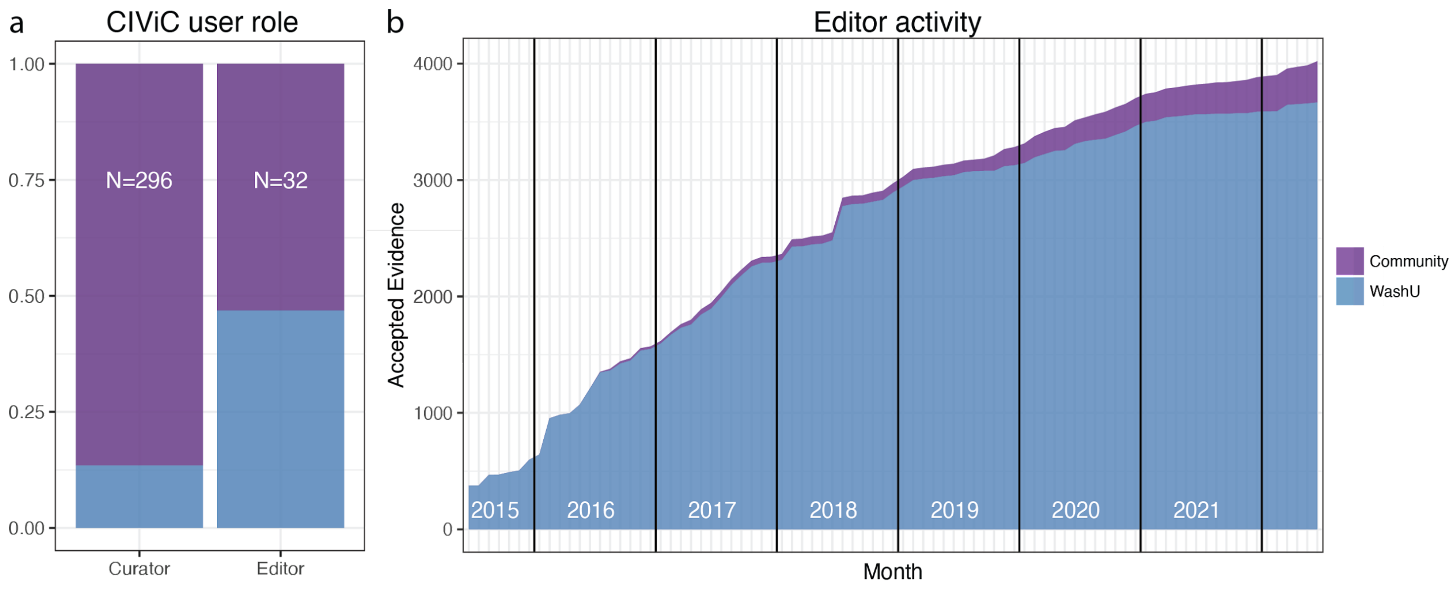

Supplemental Figure 3. Range of curation activities within CIViC

Displayed are a variety of actions that can be performed within the CIViC interface that range in time burden for Curators. Comments and flags are the least burdensome activities whereas the generation of new Evidence Items and Assertions represent high time and knowledge requirements. Other activities, including suggesting sources or revisions, can be performed with moderate burden on Curators.

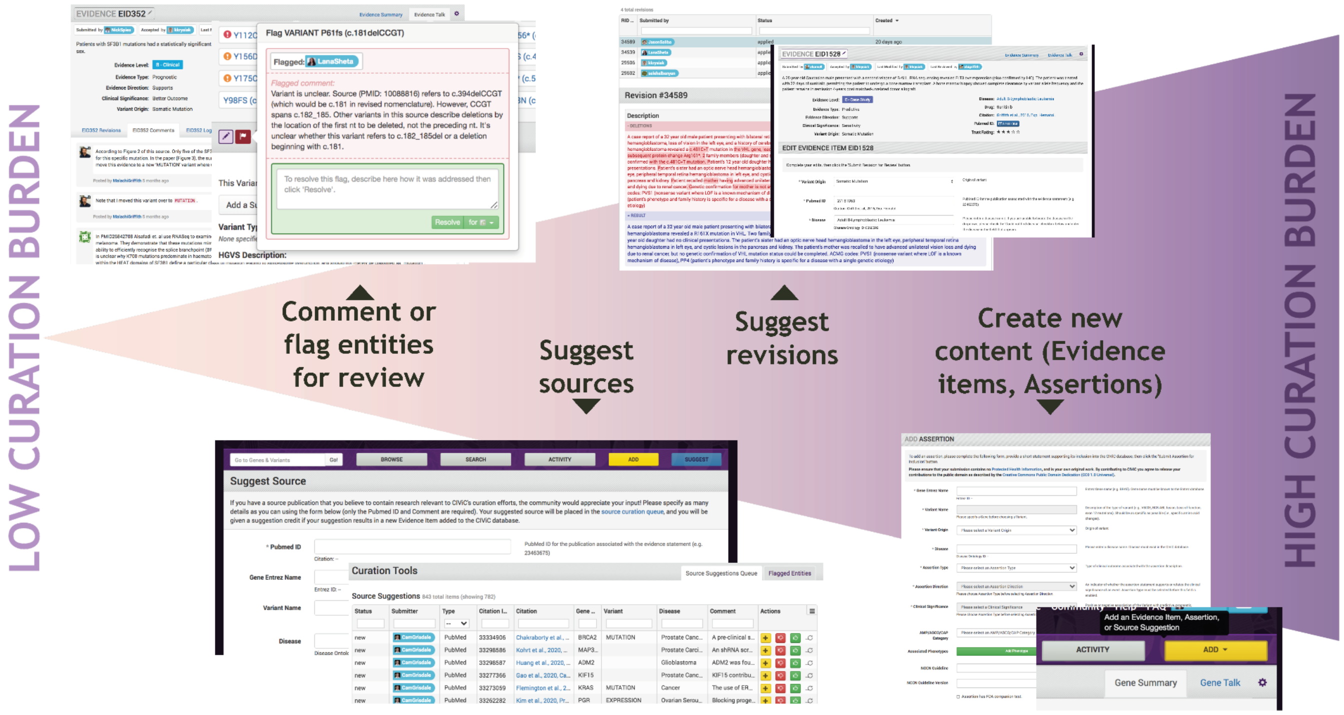

Supplemental Figure 4. Example of a CIViC Assertion

Assertions summarize a collection of CIViC Evidence Items for a given Variant and Disease. Assertions are of Predictive, Prognostic, Diagnostic, Predisposing or Oncogenic type. Assertions show provenance by linking to underlying Evidence Items, which in turn link back to primary literature sources, and Assertions show the trail of Curator submission and Editor moderation. They include a curator written Summary and Description, and show regulatory information. Assertions also show AMP/ASCO/CAP Tier (8) for Predictive, Prognostic and Diagnostic types, ACMG/AMP(9) pathogenicity evaluation for the Predisposing Assertion Type, and ClinGen/CGC/VICC(4) oncogenicity evaluation for the Oncogenic Assertion Type.

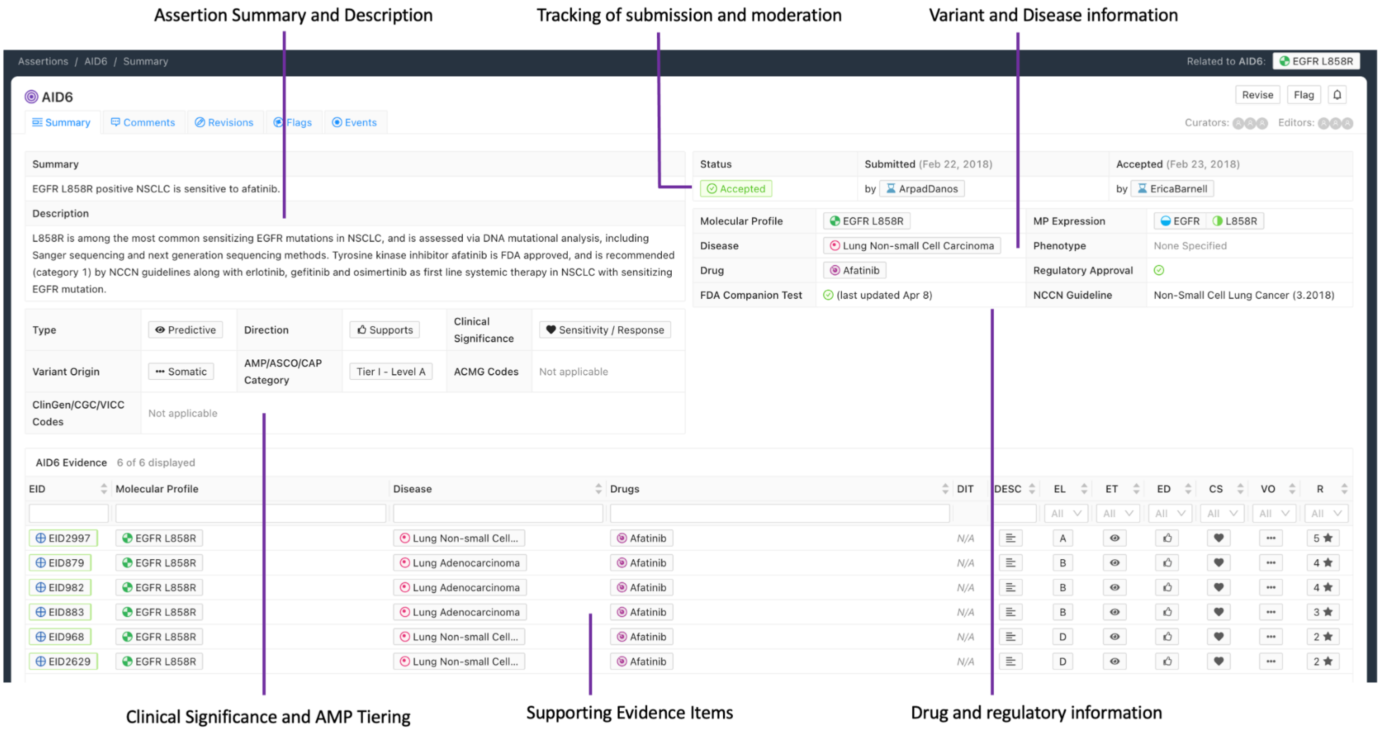

Supplemental Figure 5. Curator incentivization through Organizations and Badges

Activities attributed to CIViC Organizations including: **a)** users associated with each Organization, **b)** changes suggested by each Organization, and **c)** Badge counts for users associated with each Organization. More statistics can be found at https://civicdb.org/organizations/home.

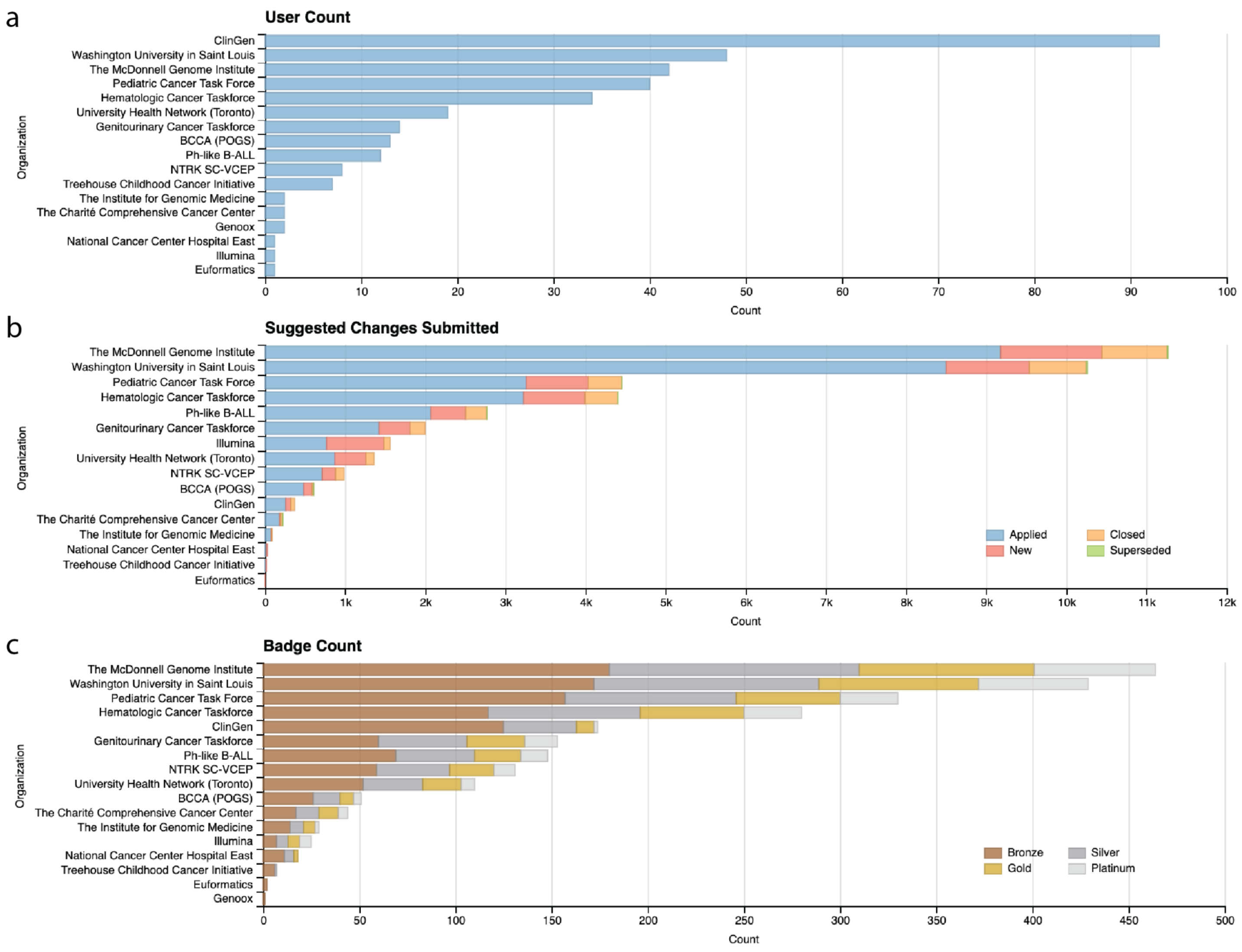

Supplemental Figure 6. CIViC Data Model

Several upgrades have been made to the CIViC interface since the introduction of the knowledgebase in 2017(10). Key updates include the introduction of Variant Groups, Assertions, Source Suggestions, and Phenotypes as well as the expansion of Evidence Types and Sources. Many of these features were implemented based on existing collaborations with CIViC users or to align CIViC curation with recognized guidelines.

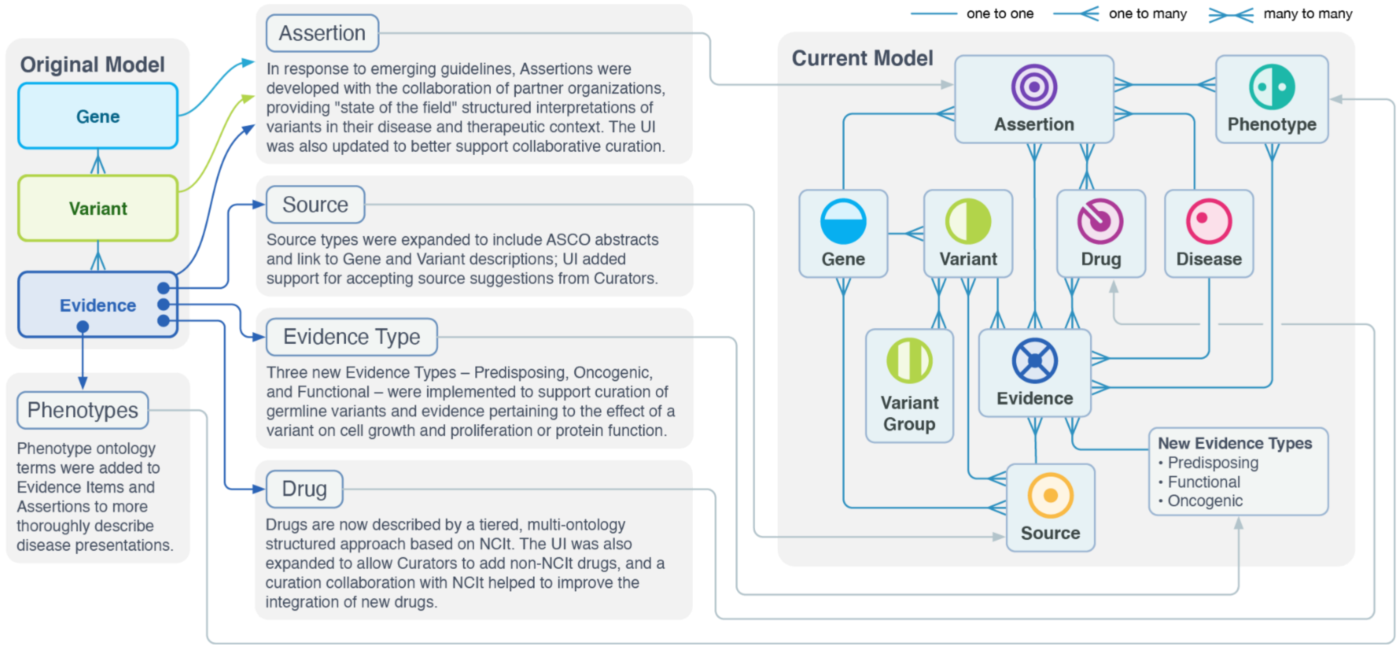

Supplemental Figure 7. Germline and somatic *VHL* variants with Evidence Items and associated Human Phenotype Ontology terms

CIViC Evidence Items include *VHL* variants identified in either somatic (top) or germline (bottom) contexts and are displayed here using St Jude's ProteinPaint(11). Numbers represent the total Evidence Item counts (submitted or accepted) associated with each Variant. Each Evidence Item can have more than one HPO term associated, the distribution of these terms is displayed in gray with bars representing their relative frequency. Only small Variants (single nucleotide variants and small insertion / deletions) with curated coordinates are shown.

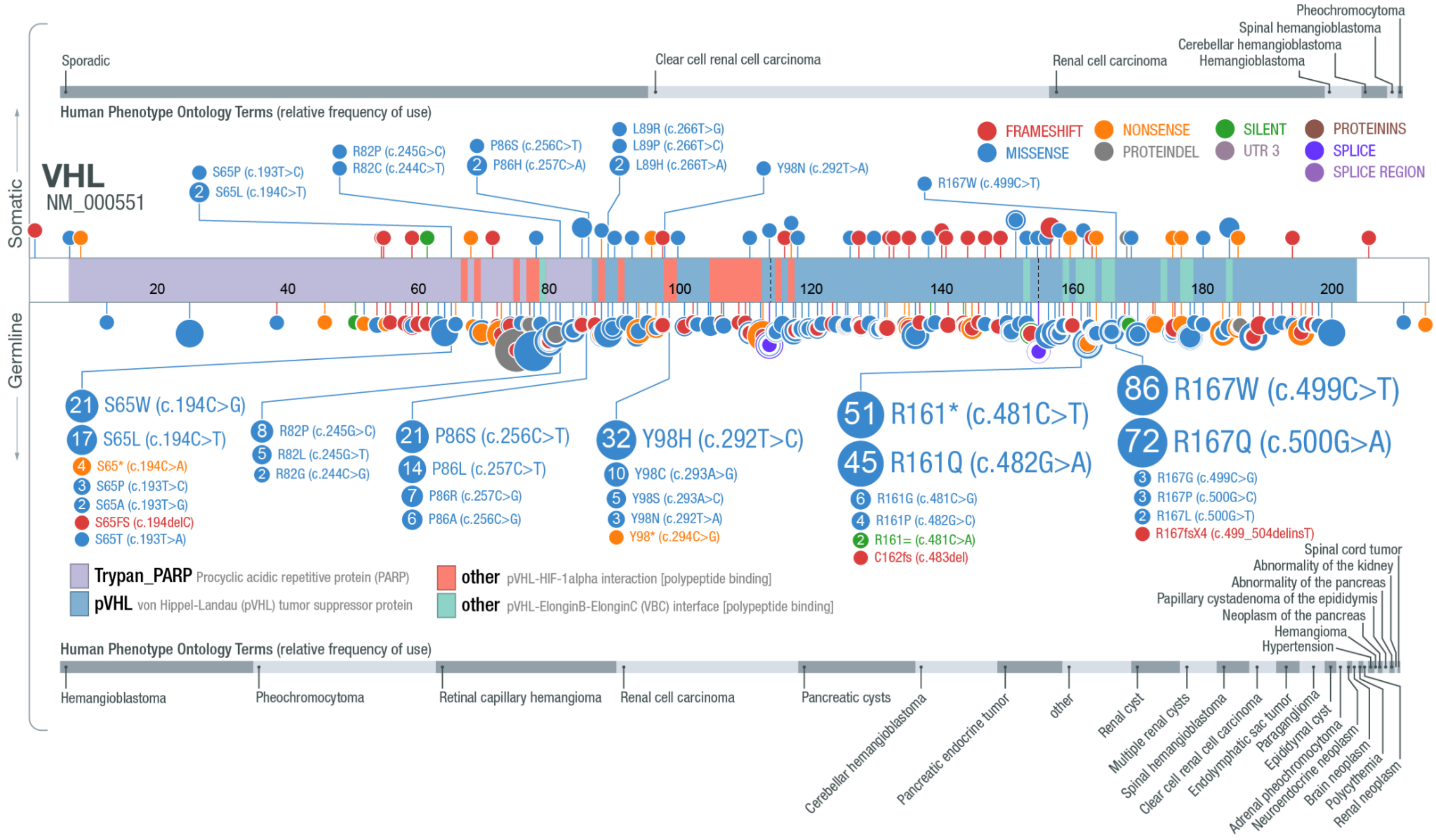

Supplemental Figure 8. New ‘Add Drug’ Interface

The NCI Thesaurus (NCIt) ontology is used to normalize Drug Names and Drug Aliases in CIViC. **a)** All Drugs in CIViC and the imported NCIt subset* are searchable when entering or editing Drug Names in CIViC. Evidence Items may also be associated with more than one Drug that is used as Substitute, Combination or Sequential treatments in a trial or experiment. **b)** Not all experimental compounds and Drug Aliases needed by CIViC Curators are in NCIt. In order to capture these terms, users are still able to add drug concepts not currently in NCIt via the “Create A New Drug” form. **c)** Display for successful addition of a Drug to CIViC. This new Drug will appear capitalized, and if approved by the moderation process, will subsequently be submitted to NCIt by CIViC Curators to improve its use in both resources.

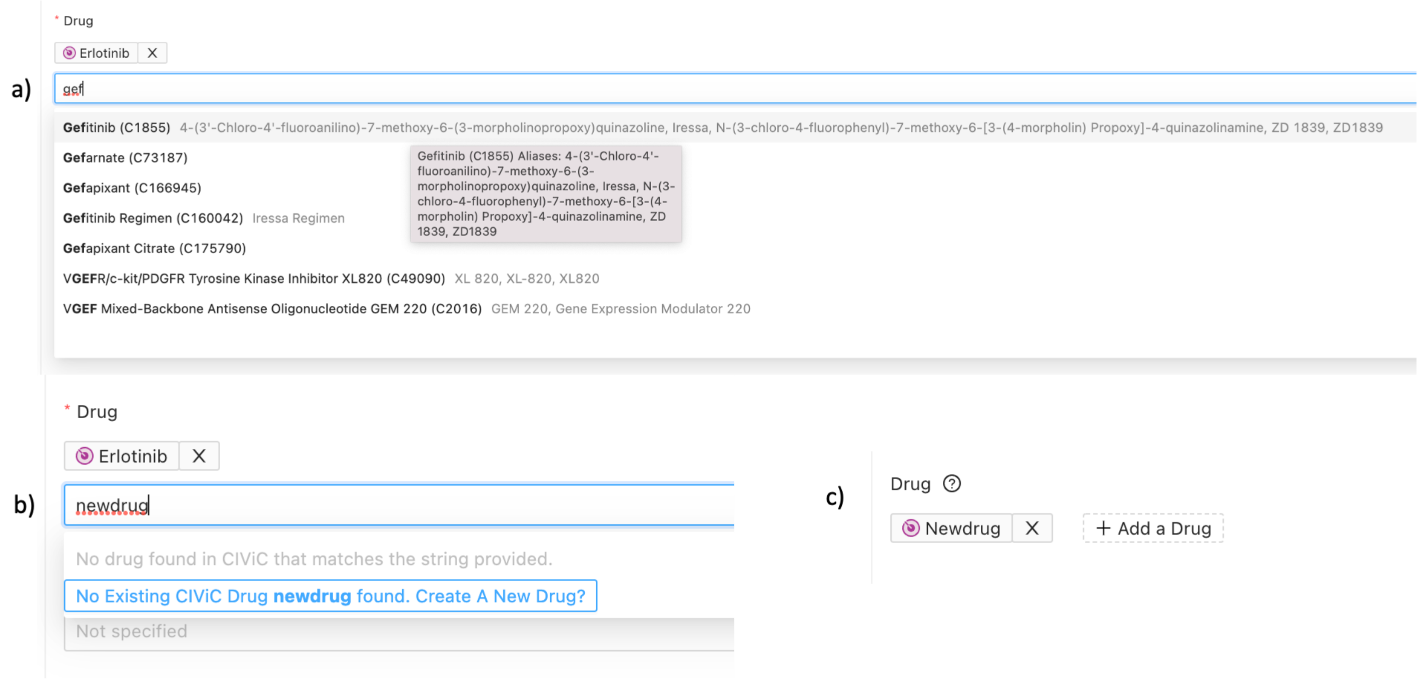

**CIViC imports the following NCIt semantic types: 'Pharmacologic Substance', 'Pharmacological Substance', 'Clinical Drug', 'Therapeutic or Preventive Procedure', 'Hazardous or Poisonous Substance'*

Supplemental Figure 9. Mapping of CIViC Functional Evidence to the classical genetic concepts of Müller's Morphs

CIViC Functional Clinical Significance annotations are derived from the classical Müller Morphs(12), represented here on a relative variant effect scale to illustrate the spectrum of clinically-relevant alterations a variant can have on protein function. These functional nuances are captured as Evidence and combined with other Evidence Types to support Assertions - variant interpretations with Predictive, Diagnostic, Prognostic, Oncogenic, or (cancer) Predisposing associations.

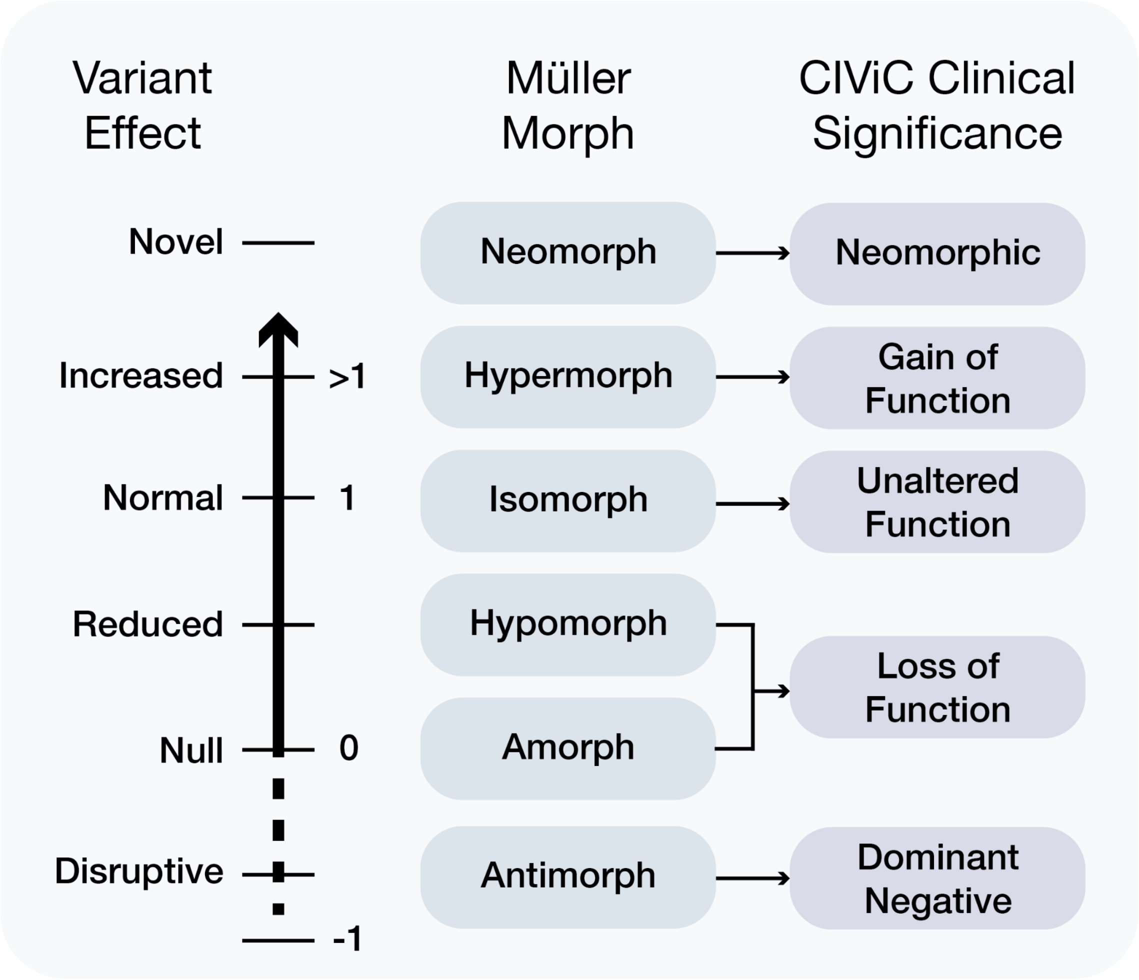

Supplemental Figure 10. Functional Evidence Item examples

Examples of curated, moderated and subsequently accepted Functional Evidence Items (EIDs) taken from the CIViC knowledgebase, as displayed in the user interface. **a)** Functional EID describing preclinical work demonstrating a gain of function effect associated with the non-canonical BRAF A728V variant. **b)** Functional EID describing a protein activity assay indicating temperature-sensitive loss of function associated with TP53 A161T. **c)** Functional EID describing preclinical assays which indicate a dominant negative effect associated with the TP53 R248Q variant.

a)
b)
c)

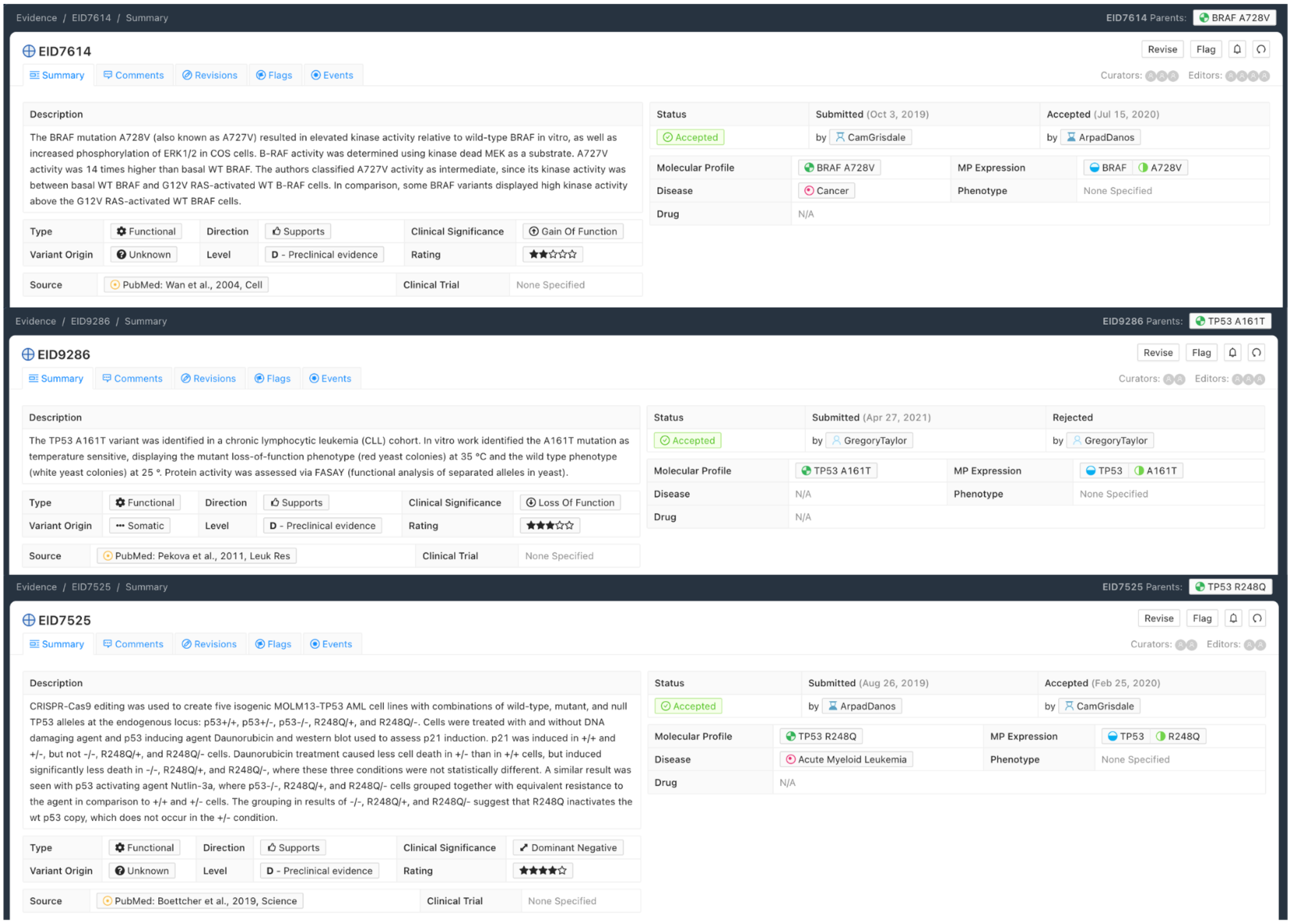

Supplemental Figure 11. Oncogenic Evidence Item examples

Examples of curated, moderated and subsequently accepted Oncogenic Evidence Items (EIDs) taken from the CIViC knowledgebase, as displayed on the user interface. **a)** Oncogenic EID describing preclinical work characterizing density-independent cell growth (foci formation) associated with KRAS Q61H transfection. **b)** Oncogenic EID describing experiments in cell lines and mice on a rare KRAS in-frame insertion variant discovered in a colorectal cancer patient cohort. The rare KRAS variant is able to promote colony formation in culture and tumor growth in nude mice comparable to a positive control KRAS G12V variant. When compared to controls (G12V and wildtype), these experiments support an oncogenic effect caused by this rare KRAS G10_A11insG variant. **c)** Oncogenic EID describing a case study with a sporadic renal cell carcinoma patient harboring a somatic *VHL* frameshift variant c.163_164delGA.

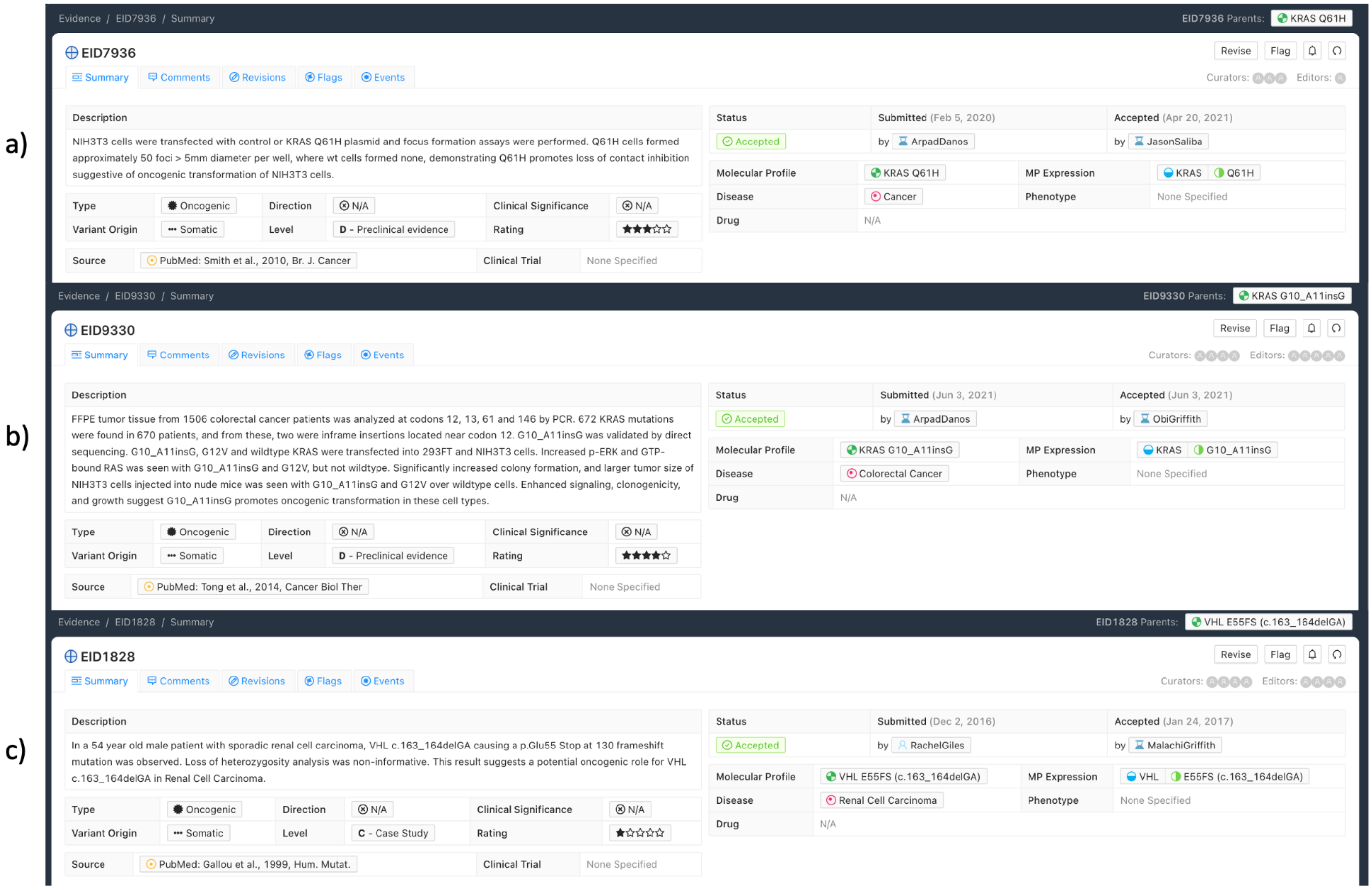

Supplemental Figure 12. Integration of Sources into CIViC over existence of the knowledgebase

a) This density plot shows monthly counts of unique Sources added, each associated with newly curated content, since the launch of the web interface. A single Source in CIViC may be associated with multiple Evidence Items (e.g., evidence for different Evidence Types or Variants). Each publication is only counted when it is first added to the CIViC knowledgebase. Peer-reviewed, PubMed-indexed literature (purple) was the first Source Type supported in CIViC. The ability to use American Society of Clinical Oncology meeting abstracts as a Source Type was launched in mid-2019 (green). Black dots indicate the accumulation of Sources in the Source Suggestion Queue. **b)** Screenshot of the Source Suggestions Queue (https://civicdb.org/curation/queues/pending-sources) where Curators can quickly recommend publications for curation. To support low-effort contributions, only the publication type (PubMed or ASCO abstract), Citation ID and a comment are required for entry. Hover text shows a comment provided by the Curator at submission. The displayed grid is filtered using type-ahead to citations of the 2010's. Additional sorting and filtering can be performed on the various displayed entities to enable quick searching for Diseases, Genes, etc of interest.

a

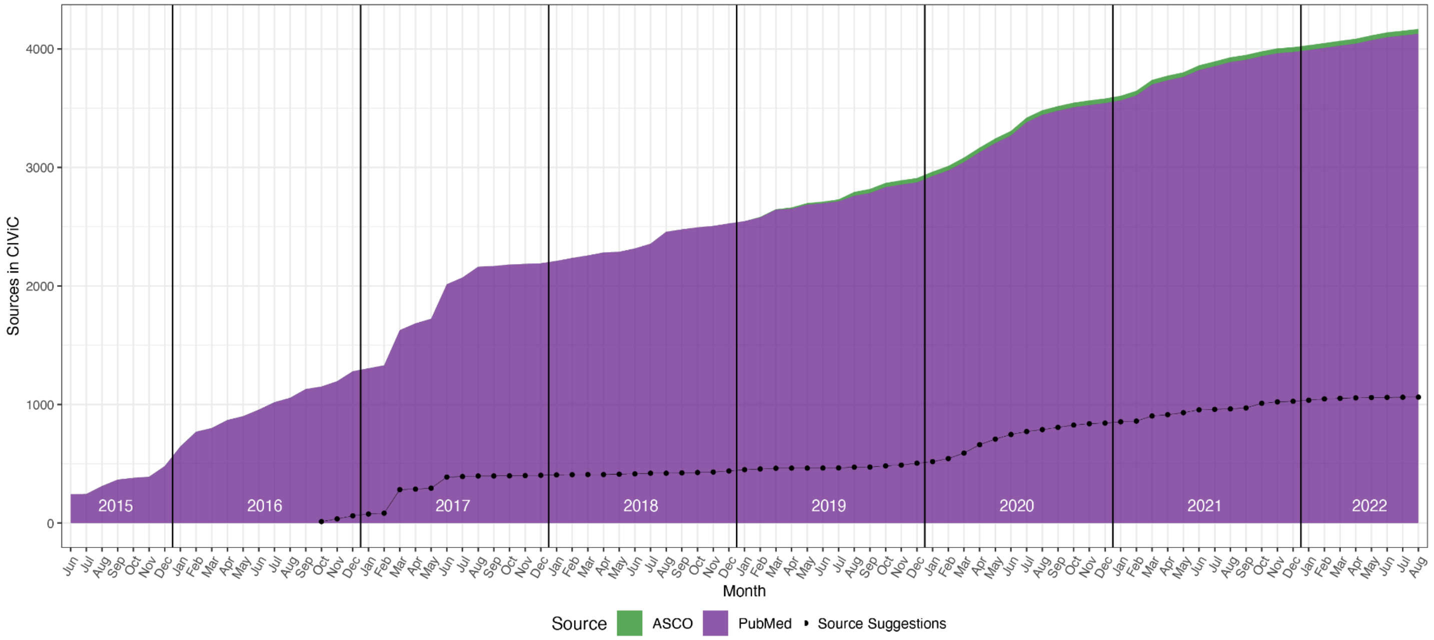

b

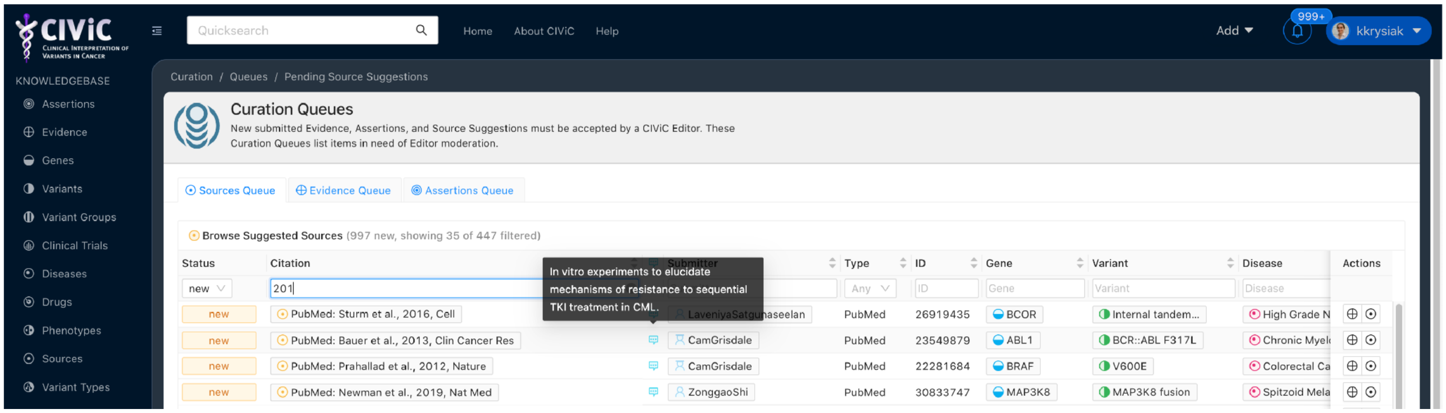

Supplemental Figure 13. Improved search capabilities

The original Main Search bar was available on all CIViC pages (**top**). This quick search allowed users to match Gene, Gene Alias, or Variant Name using a type-ahead search. Searching for specific Evidence Items or other CIViC entities required the Advanced Search page (**middle**). The new Quick Search feature of CIViC (**bottom**) available on all pages, supports elastic search with more entity types (e.g., Variant Groups and Aliases) and CIViC ID-based searching (e.g., Assertion IDs, Evidence IDs).

Main Search Bar (2016)

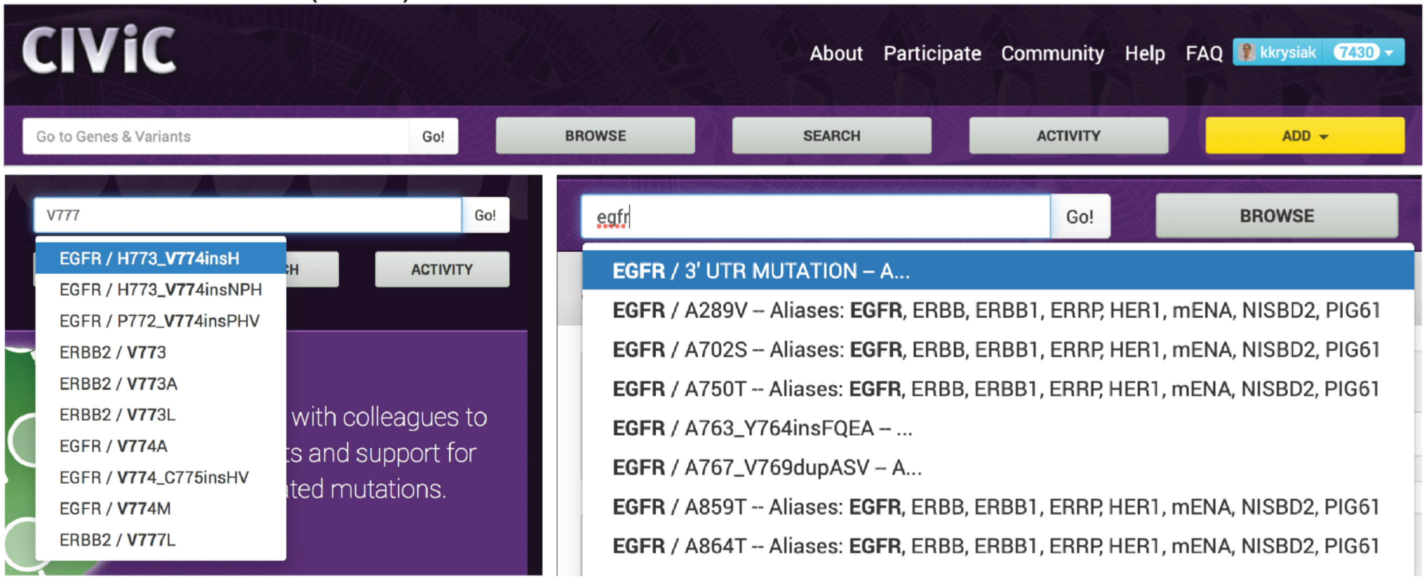

Advanced Search Page (2016)

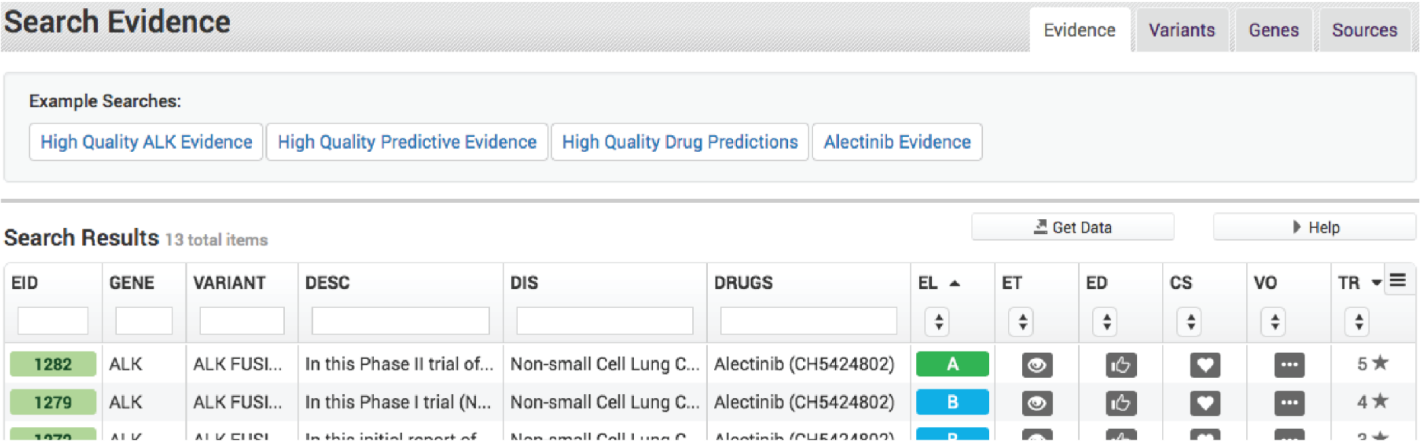

Quick Search (2022)

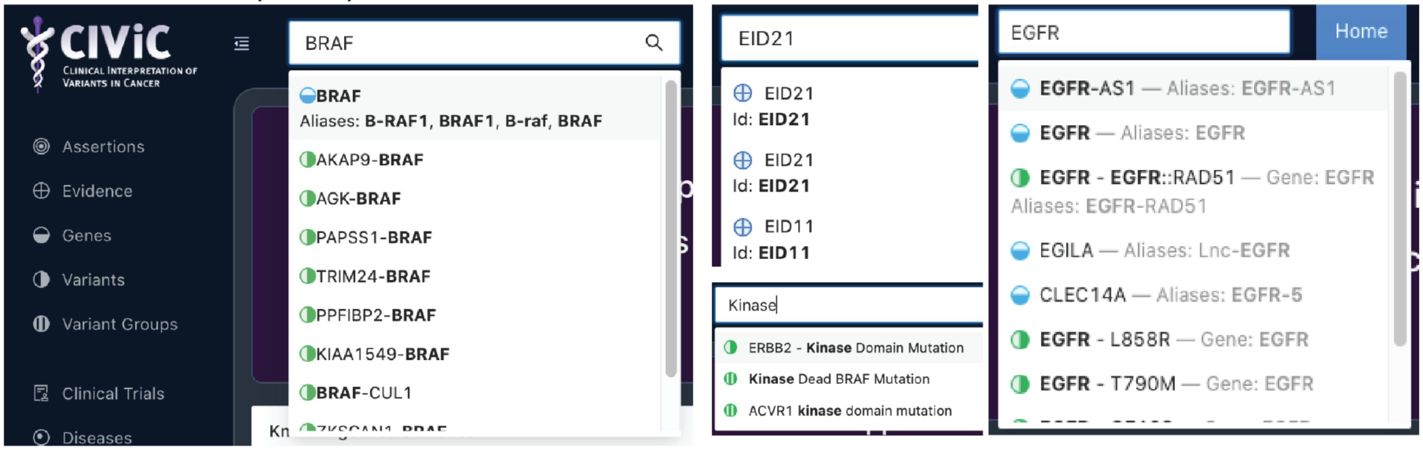

Supplemental Figure 14. Variant interface, then and now

Changes to the CIViC Variant representation in the User Interface. The original interface circa 2016(10) (top) had Variants listed to the side of Evidence and Variant Summary, which was moved to accommodate a larger number of Variants and improve searching in the current interface (bottom). **a)** Variants can be rapidly filtered by name using the quick filter feature. **b)** CIViC Variant Evidence Scores were added to weight the accumulation of Evidence relative to other CIViC Variants and support the OpenCAP design tool(13). **c)** Links to the ClinGen Allele Registry(14) are automatically generated when curated Variant Coordinates match an existing Allele Registry Allele. **d)** Curated Aliases can be added to Variant Names to enhance Variant searching throughout the UI. **e)** The Revisions tab will show all historical changes to the CIViC entity, but also alert users to outstanding, unmoderated suggested changes.

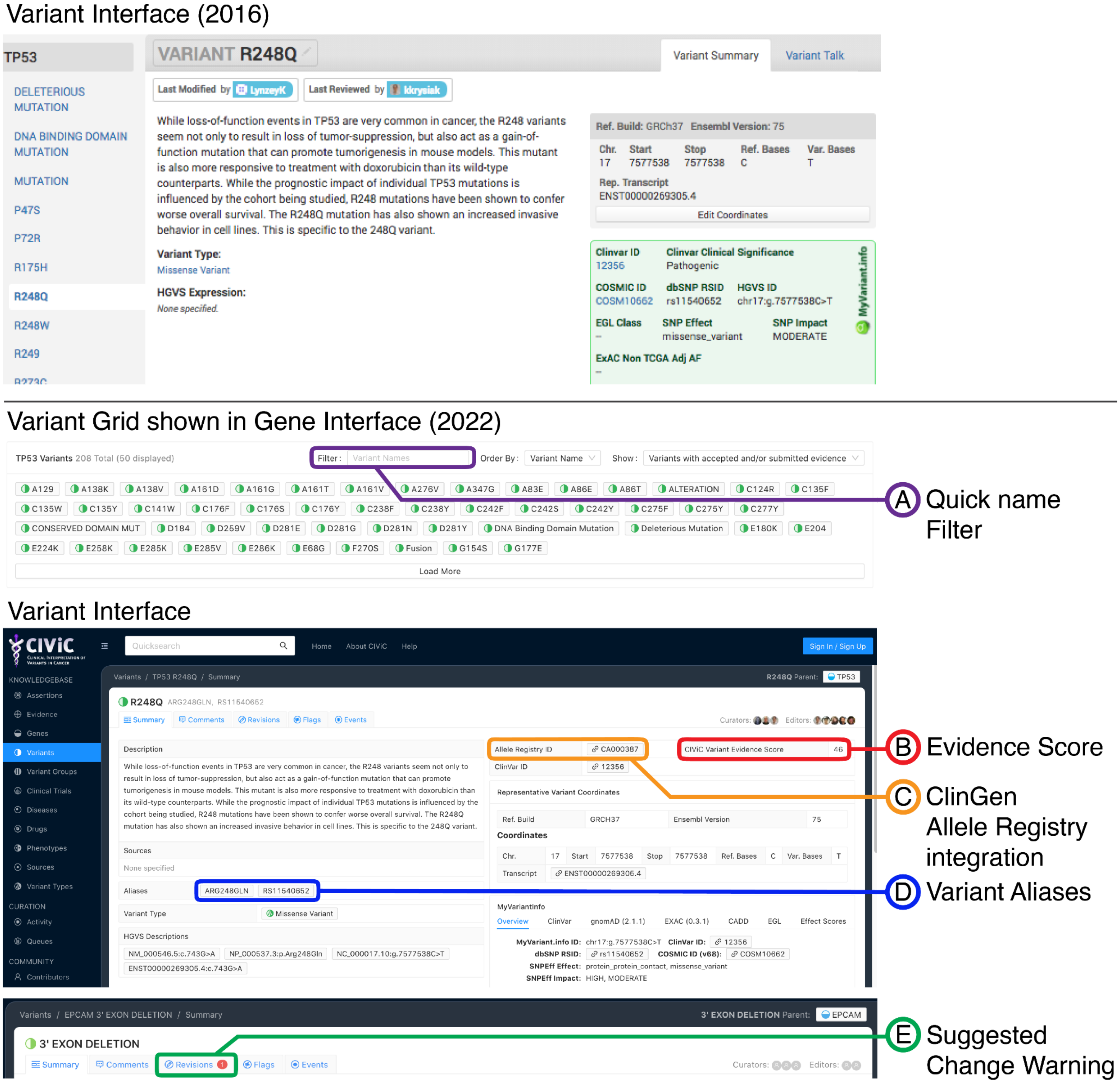

Supplementary Tables

Supplemental Table 1. Diseases associated with 10 or more accepted Evidence Items

Disease	DOID	Total Evidence	Accepted Evidence
Von Hippel-Lindau Disease	DOID:14175	2620	588
Lung Non-small Cell Carcinoma	DOID:3908	707	376
Colorectal Cancer	DOID:9256	642	337
Chronic Myeloid Leukemia	DOID:8552	476	305
Acute Myeloid Leukemia	DOID:9119	348	217
Cancer	DOID:162	275	174
Melanoma	DOID:1909	253	126
Breast Cancer	DOID:1612	253	168
Gastrointestinal Stromal Tumor	DOID:9253	225	90
Lung Adenocarcinoma	DOID:3910	175	93
Her2-receptor Positive Breast Cancer	DOID:0060079	108	84
Glioblastoma	DOID:3068	97	35
Childhood B-cell Acute Lymphoblastic Leukemia	DOID:0080146	88	39
Renal Cell Carcinoma	DOID:4450	78	29
Prostate Cancer	DOID:10283	75	33
B-lymphoblastic Leukemia/lymphoma, BCR-ABL1–like	DOID:0080650	72	57
Chronic Lymphocytic Leukemia	DOID:1040	71	45
Ovarian Cancer	DOID:2394	67	53
Skin Melanoma	DOID:8923	66	53
Pancreatic Cancer	DOID:1793	66	31
Clear Cell Renal Cell Carcinoma	DOID:4467	63	18
Head And Neck Squamous Cell Carcinoma	DOID:5520	61	49
Multiple Myeloma	DOID:9538	48	29
B-lymphoblastic Leukemia/lymphoma	DOID:0080630	46	34
Lung Cancer	DOID:1324	44	16
Neuroblastoma	DOID:769	39	24
Transitional Cell Carcinoma	DOID:2671	37	12
Stomach Cancer	DOID:10534	30	13

Acute Lymphoblastic Leukemia	DOID:9952	30	14
Cholangiocarcinoma	DOID:4947	27	18
Endometrial Cancer	DOID:1380	27	23
Sarcoma	DOID:1115	26	14
Diffuse Midline Glioma, H3 K27M-mutant	DOID:0080684	25	10
Thyroid Gland Papillary Carcinoma	DOID:3969	24	16
Myelodysplastic Syndrome	DOID:0050908	23	12
Gastric Adenocarcinoma	DOID:3717	22	18
Bladder Carcinoma	DOID:4007	20	14
Uveal Melanoma	DOID:6039	20	17
Diffuse Large B-cell Lymphoma	DOID:0050745	17	13
Pancreatic Adenocarcinoma	DOID:4074	17	11
Acute Promyelocytic Leukemia	DOID:0060318	17	11
High Grade Glioma	DOID:3070	17	10
Stomach Carcinoma	DOID:5517	16	16
Desmoid Tumor	DOID:0080366	14	12
Childhood Low-grade Glioma	DOID:0080830	14	11
Synovial Sarcoma	DOID:5485	12	12
Childhood Acute Lymphocytic Leukemia	DOID:0080144	11	10

Supplemental Table 2. Types of Variants contained in CIViC as represented by assigned Sequence Ontology terms

Variant Type	SOID	Total Evidence	Accepted Evidence
Missense Variant	SO:0001583	4088	1716
Transcript Fusion	SO:0001886	1064	723
Not Applicable*	N/A	446	346
Transcript Variant	SO:0001576	437	250
Inframe Variant	SO:0001650	373	172
Frameshift Variant	SO:0001589	289	139
Structural Variant	SO:0001537	268	195
Protein Altering Variant	SO:0001818	257	208
Stop Gained	SO:0001587	242	85
Loss Of Function Variant	SO:0002054	242	193
Gain Of Function Variant	SO:0002053	115	99
Gene Variant	SO:0001564	95	74
Other**	-	67	48
Synonymous Variant	SO:0001819	45	21
UTR Variant	SO:0001622	13	10
Wild Type	SO:0000817	12	11
Loss Of Heterozygosity	SO:0001786	10	9

To provide a representative sample of the breadth of variant types with Evidence in CIViC, Sequence Ontology terms were collapsed into selected representative shared parent terms and a count of evidence associated was calculated. (i.e., Variants labeled with copy_number_change SO:0001563 and transcript_amplification SO:0001889 were simplified into the parent term structural_variant SO:0001537 and Evidence associated with either was summed).

**As described in the CIViC documentation, some Variants fall outside the scope of the Sequence Ontology (e.g., variants involving expression levels, epigenetic variants, etc.). These have been curated with a tag of ‘Not Applicable’ to illustrate that they have been reviewed.*

***Sequence Ontology terms that are not child terms of those listed in this table were summarized into this category including overly general parent terms (e.g., transcription_variant SO:0001549) and less frequent specific terms (e.g., dominant_negative_variant SO:0002052).*

Supplemental Table 3. Peer-reviewed publications associated with CIViC collaborations

Project	Organization	Title	PubMed ID (Ref)	Year
CIViC original publication	WashU	*CIViC is a community knowledgebase for expert crowdsourcing the clinical interpretation of variants in cancer*	28138153 (10)	2017
Standards for cancer variant interpretation and sharing	Multi-institution initiative / ClinGen Somatic Working Group	*ClinGen Cancer Somatic Working Group - standardizing and democratizing access to cancer molecular diagnostic data to drive translational research*	29218886 (3)	2018
MVLD	Multi-institution initiative / ClinGen Somatic Working Group	*Adapting crowdsourced clinical cancer curation in CIViC to the ClinGen minimum variant level data community-driven standards*	30311370 (15)	2018
CIViC SOP	WashU	*Standard operating procedure for curation and clinical interpretation of variants in cancer*	31779674 (16)	2019
CIViCmine	Canada's Michael Smith Genome Sciences Centre	*Text-mining clinically relevant cancer biomarkers for curation into the CIViC database*	31796060 (17)	2019
OpenCAP	WashU	*Open-Sourced CIViC Annotation Pipeline to Identify and Annotate Clinically Relevant Variants Using Single-Molecule Molecular Inversion Probes*	31618044 (13)	2019
CIViCpy	WashU	*CIViCpy: a Python software development and analysis toolkit for the CIViC knowledgebase*	32191543 (18)	2020
Virtual Molecular Tumor Board	Multi-institution initiative / GA4GH	*Collaborative, Multidisciplinary Evaluation of Cancer Variants Through Virtual Molecular Tumor Boards Informs Local Clinical Practices*	32644817 (19)	2020
VICC	Multi-institution initiative / GA4GH	*A harmonized meta-knowledgebase of clinical interpretations of somatic genomic variants in cancer*	32246132 (20)	2020
WikiData	Multi-institution initiative	*Wikidata as a knowledge graph for the life sciences*	32180547 (21)	2020
DGIdb	WashU	*Integration of the Drug-Gene Interaction Database (DGIdb 4.0) with open crowdsource efforts*	33237278 (22)	2021
VICC oncogenicity	Multi-institution initiative / GA4GH	*Standards for the classification of pathogenicity of somatic variants in cancer (oncogenicity): Joint recommendations of Clinical Genome Resource (ClinGen), Cancer Genomics Consortium (CGC), and Variant Interpretation for Cancer Consortium (VICC)*	35101336 (4)	2022
NTRK SC-VCEP	ClinGen Somatic Working Group	*Standardized evidence-based approach for assessment of oncogenic and clinical significance of NTRK fusions*	35366592 (23)	2022
CIViC	WashU	*A community approach to the cancer-variant-interpretation bottleneck*	35624339 (24)	2022

Supplemental Table 4. Highlights of CIViC project development and community engagement since initial publication

Project Developments	Year	Community Engagements
	**2016**	1st CIViC Hackathon and Curation Jamboree/Workshop held at NKI in Amsterdam, Netherlands<br>Launch of the CIViC Twitter account (@CIVICdb)
Variant status tags added, indicating revisions on Variant of associated Evidence Items<br>Variant View updated (see **Supplemental Figure 14**)<br>Flags added, enabling Curators to alert CIViC Editors to important errors in curated entities<br>Merge Diseases and Merge Variants functionalities added to the Administrator Interface<br>gnomAD allele frequency made available in Variant View via MyVariant.Info(25)<br>Clinical trial links imported from PubMed automatically displayed on Evidence Items<br>Statistics pages created (https://civicdb.org/statistics/evidence)	**2017**	Large scale VHL curation efforts in CIViC<br>Import of Illumina curated evidence data(26)<br>CIViC included in cBioPortal(27, 28)
Assertions advanced search functionality added<br>Clinical Significance harmonized to MVLD Terms(3)<br>ACMG/AMP variant classification (P, LP, VUS, LB, B) moved from Predisposing Evidence to Assertions<br>Organizations functionality added<br>User Profile customizations added (ORCID, URL)<br>CIViC Assertion introduced to summarize collections of Evidence Items, creating "state of the field" variant interpretation<br>AMP/ASCO/CAP Level and Tier added to Predictive, Prognostic and Diagnostic Assertions<br>ACMG/AMP evidence criteria (evidence codes) added to Predisposing Assertions<br>Functional Evidence created, to capture evidence for variant-induced changes in protein function<br>Human Phenotype Ontology (HPO) terms adopted(29)<br>ASCO abstracts added as Evidence Source<br>Evidence card view added to Assertions for quick review of all underlying Evidence	**2018**	ClinGen Somatic Cancer Pediatric Taskforce formed<br>CIViC Data Available in OpenCRAVAT(30)<br>First formal external CIViC Editor training sessions held with select VHL Curators<br>First formal ClinGen curator training session held<br>CIViC YouTube channel created to share tutorial and overview videos<br>CIViCmine User Interface launched (http://bionlp.bcgsc.ca/civicmine/)<br>VHL Variant Curation Expert Panel achieves Step 1 approval<br>2nd CIViC Hackathon and Curation Jamboree/Workshop held at Scripps in La Jolla, CA<br>Bidirectional linking to the ClinGen Allele Registry (14)
Added Suggested Change advanced search<br>Organization statistics added to Community Page<br>Warning Labels added to unmoderated Evidence Items and Assertions	**2019**	CIViC Data Available in NDex(32)<br>First ClinGen Somatic Editor training sessions held

Drugs normalized to NCIt(31)<br>Extensive Documentation added (docs.civicdb.org)<br>Variant Score introduced as an aggregate metric of curated evidence, supporting OpenCAP(13)<br>Conflict of interest (COI) functionality added, with yearly update requirement for all Editors<br>Sub-Organizations functionality added<br>Dominant Negative Clinical Significance option added to Functional Evidence Items<br>Functionality added for Curator to reject own revision	**2019**	
Oncogenic Evidence Type added<br>Release of the CIViCpy tool to aid in the use of the CIViC API<br>More detailed Variant naming standards provided and normalization performed by curators<br>Functionality added for users to choose Organization for attribution of individual curation actions<br>VCF format files added to data releases (https://civicdb.org/releases)	**2020**	ClinGen Somatic Hematologic Cancer Taskforce formed<br>NTRK Somatic Cancer Variant Curation Expert Panel achieves Step 1 approval<br>First set of Assertions made publicly available in ClinVar<br>Bidirectional linking to St. Jude Cloud PeCan/Protein Paint(33)
HPO "Onset" terms (HP:0003674) added to the pediatric cancer curation protocol<br>Monthly data dumps made available via AWS OpenData (https://registry.opendata.aws/civic/)	**2021**	Pediatric Cancer Curation Advancement Subcommittee (PCCAS) formed<br>*VHL* Variant Curation Expert Panel achieves Step 2 approval<br>FLT3 Somatic Cancer Variant Curation Expert Panel achieves Step 1 approval<br>FGFR Somatic Cancer Variant Curation Expert Panel achieves Step 1 approval
CIViC V2 interface and API released	**2022**	NTRK Somatic Cancer Variant Curation Expert Panel achieves Step 2 approval<br>Histone H3 Somatic Cancer Variant Curation Expert Panel achieves Step 1 approval<br>ClinGen Solid Tumor Task Force formed<br>3rd CIViC Hackathon and Curation Jamboree/Workshop held in Saint Louis, MO

For more detailed updates see CIViC on Github: https://github.com/griffithlab/civic-v2/releases

References

1. Budd,A., Dinkel,H., Corpas,M., Fuller,J.C., Rubinat,L., Devos,D.P., Khoueiry,P.H., Förstner,K.U., Georgatos,F., Rowland,F., *et al.* (2015) Ten simple rules for organizing an unconference. *PLoS Comput. Biol.*, **11**, e1003905.

2. Nordstrom-O'Brien,M., van der Luijt,R.B., van Rooijen,E., van den Ouweland,A.M., Majoor-Krakauer,D.F., Lolkema,M.P., van Brussel,A., Voest,E.E. and Giles,R.H. (2010) Genetic analysis of von Hippel-Lindau disease. *Hum. Mutat.*, **31**, 521–537.

3. Madhavan,S., Ritter,D., Micheel,C., Rao,S., Roy,A., Sonkin,D., Mccoy,M., Griffith,M., Griffith,O.L., Mcgarvey,P., *et al.* (2018) ClinGen Cancer Somatic Working Group - standardizing and democratizing access to cancer molecular diagnostic data to drive translational research. *Pac. Symp. Biocomput.*, **23**, 247–258.

4. Horak,P., Griffith,M., Danos,A.M., Pitel,B.A., Madhavan,S., Liu,X., Chow,C., Williams,H., Carmody,L., Barrow-Laing,L., *et al.* (2022) Standards for the classification of pathogenicity of somatic variants in cancer (oncogenicity): Joint recommendations of Clinical Genome Resource (ClinGen), Cancer Genomics Consortium (CGC), and Variant Interpretation for Cancer Consortium (VICC). *Genet. Med.*, **24**, 986–998.

5. Hanahan,D. and Weinberg,R.A. (2011) Hallmarks of cancer: the next generation. *Cell*, **144**, 646–674.

6. Tong,J.H.M., Lung,R.W.M., Sin,F.M.C., Law,P.P.Y., Kang,W., Chan,A.W.H., Ma,B.B.Y., Mak,T.W.C., Ng,S.S.M. and To,K.F. (2014) Characterization of rare transforming KRAS mutations in sporadic colorectal cancer. *Cancer Biol. Ther.*, **15**, 768–776.

7. Mateo,J., Chakravarty,D., Dienstmann,R., Jezdic,S., Gonzalez-Perez,A., Lopez-Bigas,N., Ng,C.K.Y., Bedard,P.L., Tortora,G., Douillard,J.-Y., *et al.* (2018) A framework to rank genomic alterations as targets for cancer precision medicine: the ESMO Scale for Clinical Actionability of molecular Targets (ESCAT). *Ann. Oncol.*, **29**, 1895–1902.

8. Li,M.M., Datto,M., Duncavage,E.J., Kulkarni,S., Lindeman,N.I., Roy,S., Tsimberidou,A.M., Vnencak-Jones,C.L., Wolff,D.J., Younes,A., *et al.* (2017) Standards and Guidelines for the Interpretation and Reporting of Sequence Variants in Cancer: A Joint Consensus Recommendation of the Association for Molecular Pathology, American Society of Clinical Oncology, and College of American Pathologists. *J. Mol. Diagn.*, **19**, 4–23.

9. Richards,S., Aziz,N., Bale,S., Bick,D., Das,S., Gastier-Foster,J., Grody,W.W., Hegde,M., Lyon,E., Spector,E., *et al.* (2015) Standards and guidelines for the interpretation of sequence variants: a joint consensus recommendation of the American College of Medical Genetics and Genomics and the Association for Molecular Pathology. *Genet. Med.*, **17**, 405–424.

10. Griffith,M., Spies,N.C., Krysiak,K., McMichael,J.F., Coffman,A.C., Danos,A.M., Ainscough,B.J., Ramirez,C.A., Rieke,D.T., Kujan,L., *et al.* (2017) CIViC is a community knowledgebase for expert crowdsourcing the clinical interpretation of variants in cancer. *Nat. Genet.*, **49**, 170–174.

11. Zhou,X., Edmonson,M.N., Wilkinson,M.R., Patel,A., Wu,G., Liu,Y., Li,Y., Zhang,Z., Rusch,M.C., Parker,M., *et al.* (2016) Exploring genomic alteration in pediatric cancer using ProteinPaint. *Nat. Genet.*, **48**, 4–6.

12. MULLER and H. J (1932) Further studies on the nature and causes of gene mutations. *Proc. Sixth Int. Cong. Genet., Ithaca, New York, USA*, **1**, 213–255.

13. Barnell,E.K., Waalkes,A., Mosior,M.C., Penewit,K., Cotto,K.C., Danos,A.M., Sheta,L.M., Campbell,K.M., Krysiak,K., Rieke,D., *et al.* (2019) Open-Sourced CIViC Annotation Pipeline to Identify and Annotate Clinically Relevant Variants Using Single-Molecule Molecular Inversion Probes. *JCO Clin Cancer Inform*, **3**, 1–12.

14. Pawliczek,P., Patel,R.Y., Ashmore,L.R., Jackson,A.R., Bizon,C., Nelson,T., Powell,B., Freimuth,R.R., Strande,N., Shah,N., *et al.* (2018) ClinGen Allele Registry links information about genetic variants. *Hum. Mutat.*, **39**, 1690–1701.

15. Danos,A.M., Ritter,D.I., Wagner,A.H., Krysiak,K., Sonkin,D., Micheel,C., McCoy,M., Rao,S., Raca,G., Boca,S.M., *et al.* (2018) Adapting crowdsourced clinical cancer curation in CIViC to the ClinGen minimum variant level data community-driven standards. *Hum. Mutat.*, **39**, 1721–1732.

16. Danos,A.M., Krysiak,K., Barnell,E.K., Coffman,A.C., McMichael,J.F., Kiwala,S., Spies,N.C., Sheta,L.M., Pema,S.P., Kujan,L., *et al.* (2019) Standard operating procedure for curation and clinical interpretation of variants in cancer. *Genome Med.*, **11**, 76.

17. Lever,J., Jones,M.R., Danos,A.M., Krysiak,K., Bonakdar,M., Grewal,J.K., Culibrk,L., Griffith,O.L., Griffith,M. and Jones,S.J.M. (2019) Text-mining clinically relevant cancer biomarkers for curation into the CIViC database. *Genome Med.*, **11**, 78.

18. Wagner,A.H., Kiwala,S., Coffman,A.C., McMichael,J.F., Cotto,K.C., Mooney,T.B., Barnell,E.K., Krysiak,K., Danos,A.M., Walker,J., *et al.* (2020) CIViCpy: A Python Software Development and Analysis Toolkit for the CIViC Knowledgebase. *JCO Clinical Cancer Informatics*.

19. Rao,S., Pitel,B., Wagner,A.H., Boca,S.M., McCoy,M., King,I., Gupta,S., Park,B.H., Warner,J.L., Chen,J., *et al.* (2020) Collaborative, Multidisciplinary Evaluation of Cancer Variants Through Virtual Molecular Tumor Boards Informs Local Clinical Practices. *JCO Clin Cancer Inform*, **4**, 602–613.

20. Wagner,A.H., Walsh,B., Mayfield,G., Tamborero,D., Sonkin,D., Krysiak,K., Deu-Pons,J., Duren,R.P., Gao,J., McMurry,J., *et al.* (2020) A harmonized meta-knowledgebase of clinical interpretations of somatic genomic variants in cancer. *Nat. Genet.*, **52**, 448–457.

21. Waagmeester,A., Stupp,G., Burgstaller-Muehlbacher,S., Good,B.M., Griffith,M., Griffith,O.L., Hanspers,K., Hermjakob,H., Hudson,T.S., Hybiske,K., *et al.* (2020) Wikidata as a knowledge graph for the life sciences. *Elife*, **9**.

22. Freshour,S.L., Kiwala,S., Cotto,K.C., Coffman,A.C., McMichael,J.F., Song,J.J., Griffith,M., Griffith,O.L. and Wagner,A.H. (2021) Integration of the Drug-Gene Interaction Database (DGIdb 4.0) with open crowdsource efforts. *Nucleic Acids Res.*, **49**, D1144–D1151.

23. Saliba,J., Church,A.J., Rao,S., Danos,A., Furtado,L.V., Laetsch,T., Zhang,L., Nardi,V., Lin,W.-H., Ritter,D.I., *et al.* (2022) Standardized evidence-based approach for assessment of oncogenic and clinical significance of NTRK fusions. *Cancer Genet.*, **264-265**, 50–59.

24. Krysiak,K., Danos,A.M., Kiwala,S., McMichael,J.F., Coffman,A.C., Barnell,E.K., Sheta,L., Saliba,J., Grisdale,C.J., Kujan,L., *et al.* (2022) A community approach to the cancer-variant-interpretation bottleneck. *Nat Cancer*, **3**, 522–525.

25. Xin,J., Mark,A., Afrasiabi,C., Tsueng,G., Juchler,M., Gopal,N., Stupp,G.S., Putman,T.E., Ainscough,B.J., Griffith,O.L., *et al.* (2016) High-performance web services for querying gene and variant annotation. *Genome Biol.*, **17**, 91.

26. Illumina and American Society of Clinical Oncology, Inc. Jointly Donate Thousands of Somatic Interpretations to CIViC to Advance Genomic Medicine (2017). Retrieved August 15th, 2022 from https://www.asco.org/about-asco/press-center/news-releases/illumina-and-american-society-clinical-oncology-inc-jointly

27. Cerami,E., Gao,J., Dogrusoz,U., Gross,B.E., Sumer,S.O., Aksoy,B.A., Jacobsen,A., Byrne,C.J., Heuer,M.L., Larsson,E., *et al.* (2012) The cBio cancer genomics portal: an open platform for exploring multidimensional cancer genomics data. *Cancer Discov.*, **2**, 401–404.

28. Gao,J., Aksoy,B.A., Dogrusoz,U., Dresdner,G., Gross,B., Sumer,S.O., Sun,Y., Jacobsen,A., Sinha,R., Larsson,E., *et al.* (2013) Integrative analysis of complex cancer genomics and clinical profiles using the cBioPortal. *Sci. Signal.*, **6**, l1.

29. Köhler,S., Gargano,M., Matentzoglu,N., Carmody,L.C., Lewis-Smith,D., Vasilevsky,N.A., Danis,D., Balagura,G., Baynam,G., Brower,A.M., *et al.* (2021) The Human Phenotype Ontology in 2021. *Nucleic Acids Res.*, **49**, D1207–D1217.

30. Pagel,K.A., Kim,R., Moad,K., Busby,B., Zheng,L., Tokheim,C., Ryan,M. and Karchin,R. (2020) Integrated Informatics Analysis of Cancer-Related Variants. *JCO Clin Cancer Inform*, **4**, 310–317.

31. Fragoso,G., de Coronado,S., Haber,M., Hartel,F. and Wright,L. (2004) Overview and utilization of the NCI thesaurus. *Comp. Funct. Genomics*, **5**, 648–654.

32. Pratt,D., Chen,J., Pillich,R., Rynkov,V., Gary,A., Demchak,B. and Ideker,T. (2017) NDEx 2.0: A Clearinghouse for Research on Cancer Pathways. *Cancer Res.*, **77**, e58–e61.

33. McLeod,C., Gout,A.M., Zhou,X., Thrasher,A., Rahbarinia,D., Brady,S.W., Macias,M., Birch,K., Finkelstein,D., Sunny,J., *et al.* (2021) St. Jude Cloud: A Pediatric Cancer Genomic Data-Sharing Ecosystem. *Cancer Discov.*, **11**, 1082–1099.
